# Comparative Metabolomics Reveals the Microenvironment of Common T-Helper Cells and Differential Immune Cells Linked to Unique Periapical Lesions

**DOI:** 10.3389/fimmu.2021.707267

**Published:** 2021-09-03

**Authors:** Alaa Muayad Altaie, Thenmozhi Venkatachalam, Lakshman P. Samaranayake, Sameh S. M. Soliman, Rifat Hamoudi

**Affiliations:** ^1^Research Institute for Medical and Health Sciences, University of Sharjah, Sharjah, United Arab Emirates; ^2^Department of Oral and Craniofacial Health Sciences, College of Dental Medicine, University of Sharjah, Sharjah, United Arab Emirates; ^3^Department of Clinical Sciences, College of Medicine, University of Sharjah, Sharjah, United Arab Emirates; ^4^Department of Oral Biosciences, Faculty of Dentistry, University of Hong Kong, Hong Kong, Hong Kong, SAR China; ^5^Department of Medicinal Chemistry, College of Pharmacy, University of Sharjah, Sharjah, United Arab Emirates; ^6^Division of Surgery and Interventional Science, University College London, London, United Kingdom

**Keywords:** periapical lesions, metabolomics profiling, immunological population, healthy pulp, periapical abscess, radicular cyst, periapical granuloma, gene

## Abstract

Periapical abscesses, radicular cysts, and periapical granulomas are the most frequently identified pathological lesions in the alveolar bone. While little is known about the initiation and progression of these conditions, the metabolic environment and the related immunological behaviors were examined for the first time to model the development of each pathological condition. Metabolites were extracted from each lesion and profiled using gas chromatography-mass spectrometry in comparison with healthy pulp tissue. The metabolites were clustered and linked to their related immune cell fractions. Clusters I and J in the periapical abscess upregulated the expression of MMP-9, IL-8, CYP4F3, and VEGF, while clusters L and M were related to lipophagy and apoptosis in radicular cyst, and cluster P in periapical granuloma, which contains L-(+)-lactic acid and ethylene glycol, was related to granuloma formation. Oleic acid, 17-octadecynoic acid, 1-nonadecene, and L-(+)-lactic acid were significantly the highest unique metabolites in healthy pulp tissue, periapical abscess, radicular cyst, and periapical granuloma, respectively. The correlated enriched metabolic pathways were identified, and the related active genes were predicted. Glutamatergic synapse (16–20),-hydroxyeicosatetraenoic acids, lipophagy, and retinoid X receptor coupled with vitamin D receptor were the most significantly enriched pathways in healthy control, abscess, cyst, and granuloma, respectively. Compared with the healthy control, significant upregulation in the gene expression of *CYP4F3*, *VEGF*, *IL-8*, *TLR2* (*P *< 0.0001), and *MMP-9* (*P *< 0.001) was found in the abscesses. While *IL-12A* was significantly upregulated in cysts (*P *< 0.01), *IL-17A* represents the highest significantly upregulated gene in granulomas (*P *< 0.0001). From the predicted active genes, CIBERSORT suggested the presence of natural killer cells, dendritic cells, pro-inflammatory M1 macrophages, and anti-inflammatory M2 macrophages in different proportions. In addition, the single nucleotide polymorphisms related to *IL-10*, *IL-12A*, and *IL-17D* genes were shown to be associated with periapical lesions and other oral lesions. Collectively, the unique metabolism and related immune response shape up an environment that initiates and maintains the existence and progression of these oral lesions, suggesting an important role in diagnosis and effective targeted therapy.

## Introduction

Periapical lesions are among the most frequently occurring pathological lesions (1) in the alveolar tissues surrounding the apex of the tooth root, the periodontal membrane, and the alveolar bone (2). They are predominantly precipitated by pulpal inflammation and/or necrosis, with consequent inflammatory mediators diffusing through the apical foramen into the surrounding tissues (3). Although the classification of the World Health Organization does not specifically feature the periapical lesions (4), Nair has classified periapical radiolucency into apical abscess (acute or chronic), acute apical periodontitis (primary or secondary), periapical cyst (true or pocket), and chronic apical periodontitis (granuloma) (5).

Periapical abscess is a localized collection of pus that occurs at the end of a root apex (6). Detectable levels of IL-8 are found in approximately 95% of periapical exudates, suggesting a pivotal role of IL-8 in acute phases of apical disease (7). Endodontic microorganisms are able to induce the production of IL-8 by pulp fibroblasts and osteoblasts (8). It has been found that leukotriene B4 (LTB4) is formed when arachidonic acid is oxidized *via* the lipoxygenase pathway, causing adhesion of polymorphonuclear leukocytes (PMNs) to the endothelial walls that attract macrophages to the affected area, leading to severe damage to the host tissues (9).

Radicular cyst is the most common odontogenic cyst (10) associated with bacterial (11), fungal, archaeal, and viral infections (12). A high level of bacterial endotoxin was detected in radicular cysts and was found to induce a proliferative effect on the epithelial cells (13). While the role of IL-12 (14) in the pathogenesis of radicular cyst is controversial, a confirmative role of IL-17 (15) on the immune response and bone resorption has been noted.

Periapical granuloma is a chronic inflammation located at the apex of a non-vital tooth. It comprises granulation and scar tissues permeated by diverse inflammatory cells including lymphocytes, plasma cells, macrophages, and mast cells (16). IL-17 (17), IL-10 (18), and IL-6 (19) are important cytokines found in apical periodontitis. The granulomatous tissue may frequently harbor microbes including bacteria (11), fungi, archaea, and viruses (12). The type of immune response in the periapical lesions is thought to be determined by the apically resident bacterial phylotypes (20).

There is a close relationship between the pathology of the periapical abscess, the resultant granuloma, and the eventual cystic lesion. The abscess theory describes the conversion of an acute inflammatory abscess to an inflammatory periapical cyst by enclosing and delimiting the area with proliferating epithelia (21). On the other hand, a periapical cyst may evolve from periapical granuloma as a consequence of untreated chronic periapical periodontitis (22). Furthermore, a dormant periapical abscess may develop into a periapical granuloma (1). Understanding this pathophysiological transformation as well as the metabolic behavior of the causative bacteria and their effect on the host responses can now be accomplished using new generation molecular methods. Furthermore, the metabolic background underlying these conditions or their roles in lesion progression have never been studied.

Distinct subpopulations of inflammatory cells have been described in periapical lesions. T cells are abundant in periapical lesions, but the activation and function of these cells are not well understood. T-helper cells are known to be mediators of bone resorption, while a larger population of activated T cells was found in granulomas compared with cysts (23). The cellular component of the periapical abscess is predominantly neutrophils and macrophages (24), although a scanty population of dendritic cells has been observed in periapical granulomas and cysts (25).

Metabolomics refer to all metabolites present in a given biological system, fluid, cell, or tissue (26). Metabolic profiling is highly informative since metabolites act as the end products of any biochemical metabolic pathway (27). Such pathways in immune cellular functions may be seen as surrogate markers of inflammation (28) consequential to host–microbe interactions (29). However, metabolite profiling in relation to dentoalveolar lesions has never been attempted before. In this study, the unique metabolites related to periapical lesions in comparison with healthy pulp tissue were mapped along with enriched pathways and then linked to previously published data involving the related active genes. The combined data generate a significant association between the metabolic pattern, enriched pathways, and the existence, maintenance, and progression of dentoalveolar lesions. Such data obtained based on metabolomics and immunological behavior can be used to decipher the transitional pathogenesis between periapical lesions.

## Materials and Methods

### Subject Population and Clinical Examination

This study includes 58 samples, consisting of 37 males and 21 females, with an age range between 20 and 66 years old. The samples were 15 periapical abscesses, 14 radicular cysts, 14 preapical granulomas, and 15 healthy pulp tissues. Clinical and dental characteristics of the patients included in the metabolomics and gene expression analyses are reported in **Table 1**. The inclusion criteria for the periapical lesions were teeth with carious lesions, necrotic pulps, evidence of periradicular radiolucency, and bone loss, in addition to facial pain and swelling in cases of periapical abscess. The general exclusion criteria were systemic disease, disorder affecting immunity, corticosteroid treatment, pregnancy, radiotherapy to the head or neck region, tooth mobility, and vertical tooth fracture.

**Table 1:** Clinical characteristics of the tissue samples involved in metabolomics and gene expression analyses.

Sample	Sex	Age (years)	Tooth	Lesion/clinical characteristics	Experimental method
Healthy control 1	Female	28	48	Completely impacted tooth	GC-MS
Healthy control 2	Female	28	18	Completely impacted tooth	GC-MS
Healthy control 3	Male	65	48	Completely impacted tooth	GC-MS
Healthy control 4	Female	31	48	Completely impacted tooth	GC-MS
Healthy control 5	Female	22	48	Completely impacted tooth	GC-MS
Healthy control 6	Female	28	18	Completely impacted tooth	qRT-PCR
Healthy control 7	Female	28	48	Completely impacted tooth	qRT-PCR
Healthy control 8	Male	60	48	Completely impacted tooth	qRT-PCR
Healthy control 9	Female	31	48	Completely impacted tooth	qRT-PCR
Healthy control 10	Male	24	48	Completely impacted tooth	qRT-PCR
Healthy control 11	Male	20	38	Completely impacted tooth	qRT-PCR
Healthy control 12	Female	22	38	Completely impacted tooth	qRT-PCR
Healthy control 13	Female	35	28	Completely impacted tooth	qRT-PCR
Healthy control 14	Female	22	48	Completely impacted tooth	qRT-PCR
Healthy control 15	Female	28	38	Completely impacted tooth	qRT-PCR
Periapical abscess 1	Male	66	37	Swelling/pain/root resorption	GC-MS
Periapical abscess 2	Male	40	27	Swelling/pain/slight root resorption	GC-MS
Periapical abscess 3	Male	35	36	Swelling/pain/fever/root resorption	GC-MS
Periapical abscess 4	Female	54	15	Swelling/pain/slight root resorption	GC-MS
Periapical abscess 5	Female	35	35	Swelling/pain/slight root resorption	GC-MS
Periapical abscess 6	Male	25	25	Swelling/pain/slight root resorption	qRT-PCR
Periapical abscess 7	Male	30	36	Swelling/pain/slight root resorption	qRT-PCR
Periapical abscess 8	Male	40	27	Swelling/pain/slight root resorption	qRT-PCR
Periapical abscess 9	Male	45	16	Mild swelling/pain/slight root resorption	qRT-PCR
Periapical abscess 10	Male	20	45	Swelling/pain/slight root resorption	qRT-PCR
Periapical abscess 11	Male	24	46	Swelling/pain/slight root resorption	qRT-PCR
Periapical abscess 12	Male	35	15	Swelling/pain/slight root resorption	qRT-PCR
Periapical abscess 13	Male	27	17	Swelling/pain/root resorption	qRT-PCR
Periapical abscess 14	Male	52	13	Swelling/pain/slight root resorption	qRT-PCR
Periapical abscess 15	Male	34	46	Swelling/pain/slight root resorption	qRT-PCR
Radicular cyst 1	Male	24	46	~3 mm/no root resorption	GC-MS
Radicular cyst 2	Male	35	46	~4 mm/no root resorption	GC-MS
Radicular cyst 3	Female	54	43	~4 mm/no root resorption	GC-MS
Radicular cyst 4	Male	25	28	~4 mm/slight root resorption	GC-MS
Radicular cyst 5	Male	25	28	~5 mm/root resorption	qRT-PCR
Radicular cyst 6	Male	24	46	~4 mm/no root resorption	qRT-PCR
Radicular cyst 7	Male	35	46	~3 mm/no root resorption	qRT-PCR
Radicular cyst 8	Male	54	44	~3 mm/no root resorption	qRT-PCR
Radicular cyst 9	Male	35	15	~4 mm/no root resorption	qRT-PCR
Radicular cyst 10	Female	36	17	~5 mm/slight root resorption	qRT-PCR
Radicular cyst 11	Male	32	35	~4 mm/slight root resorption	qRT-PCR
Radicular cyst 12	Male	33	27	~6 mm/slight root resorption	qRT-PCR
Radicular cyst 13	Male	49	46	~6 mm/slight root resorption	qRT-PCR
Radicular cyst 14	Female	20	46	~4 mm/slight root resorption	qRT-PCR
Periapical granuloma 1	Male	37	18	~5 mm/root resorption	GC-MS
Periapical granuloma 2	Female	20	15	~3 mm/slight root resorption	GC-MS
Periapical granuloma 3	Male	49	16	~6 mm/root resorption	GC-MS
Periapical granuloma 4	Female	33	47	~3 mm/slight root resorption	GC-MS
Periapical granuloma 5	Male	25	36	~5 mm/slight root resorption	qRT-PCR
Periapical granuloma 6	Male	42	25	~4 mm/root resorption	qRT-PCR
Periapical granuloma 7	Male	49	17	~4 mm/slight root resorption	qRT-PCR
Periapical granuloma 8	Male	37	18	~6 mm/root resorption	qRT-PCR
Periapical granuloma 9	Male	38	15	~4 mm/root resorption	qRT-PCR
Periapical granuloma 10	Male	21	16	~3 mm/root resorption	qRT-PCR
Periapical granuloma 11	Female	20	15	~5 mm/root resorption	qRT-PCR
Periapical granuloma 12	Female	35	25	~4 mm/root resorption	qRT-PCR
Periapical granuloma 13	Male	49	16	~7 mm/root resorption	qRT-PCR
Periapical granuloma 14	Female	33	47	~4 mm/slight root resorption	qRT-PCR

Sampling procedure for all periapical lesions was conducted in a strict aseptic manner. Periapical abscess aspiration was performed as described previously (30), while after tooth extraction, periapical cyst and granuloma were isolated using a sterile surgical scalpel blade No. 11, rinsed with sterile normal saline to remove the planktonic bacteria and blood. All samples were stored at −80°C and part of the periapical cyst and granuloma was fixed in 10% formalin for histopathological analysis.

Teeth of healthy pulp tissue samples were used as healthy control and collected after surgical extraction of impacted wisdom teeth (third molar teeth). The included teeth were free of caries, periodontal disease, and without any fracture or destruction of the tooth during the surgical extraction. The exclusion criteria included systemic disease, disorder affecting immunity, corticosteroid treatment, pregnancy, and radiotherapy to the head or neck region. After surgical extraction procedure was performed and the periodontal tissues were scraped, tooth surface was then cleaned with povidone-iodine solution and 70% ethanol to prevent contamination from oral bacteria and washed five times with sterile normal saline. A modified procedure was performed in isolating the dental pulp (31). When a thin layer of dentin appears, a sterile spoon excavator was used to remove this layer of dentin to avoid any further heating to the pulp. A sterile barbed broach gauge No. 60 was used to pick up the dental pulp and stored at −80°C.

### Sample Size Calculation

In order to determine if the cohort used in this study has sufficient power to allow for the identification of key biomarkers involved in the microenvironment of periapical lesions, sample size calculation was carried out based on other studies (32, 33). Since periapical lesions are different from other chronic inflammatory diseases, access to periapical lesion material is more limiting; hence, we carried out metabolomics screening followed by validation of the expression of related target genes using quantitative real-time PCR (qRT-PCR). Thus, for whole metabolomics screening, the standard deviation for the detection of activated metabolites is determined to be around 1.5 (*σ* = 1.5) and the effect size around 4 as the periapical lesions are generally well characterized. Power calculations set with *P*-value = 0.05 (5% significant testing) and power of 95% using R (version 3.6.2) showed that the minimum required number of samples per group was four (32). Once the activated metabolites are determined, related expression of the genes involved with them is measured using qRT-PCR.

For the targeted approach of measuring gene expression using qRT-PCR, the sample size is generally bigger than that for screening because the number of targets will be less. Thus, the standard deviation is determined to be 1 (*σ* = 1) and the effect size around 1.5. Taking those parameters into account together with *P*-value = 0.05 (5% significant testing) and power of 90%, power calculations in R showed that the minimum required number of samples per group was 10 (33).

Taken together, the sample size calculation for this study was met, as **Table 1** shows that all metabolomics study had a minimum of four samples per group and qRT-PCR had 10 samples per group, respectively.

### Histopathological Analysis

The retrieved histological specimens of radicular cyst and periapical granuloma were fixed in 10% neutral buffered formalin and subsequently embedded within paraffin wax and cut into 3 µm thick sections using a microtome (Thermo Fisher Scientific, Santa Clara, CA, USA). The histopathology of the lesions was assessed by a traditional hematoxylin and eosin staining. The differentiation between a radicular cyst and a periapical granuloma was made by a qualified pathologist.

### Sample Preparation for Gas Chromatography-Mass Spectrometry Analysis

Metabolite extraction was performed using chloroform. Approximately 600 mg of each sample was used. All samples were mixed with 500 µl of chloroform (Fisher Scientific), followed by water bath sonication at room temperature for 30 min. The samples were then homogenized in the same solvent using sterile pestles (Axygen Scientific, Union City, CA, USA). The supernatants were collected by filtration and the chloroform was evaporated. The dried residues were derivatized by adding 70 µl of N-trimethylsilyl-N-methyl trifluoroacetamide and trimethylchlorosilane (MSTFA + 1% TMS) followed by vortexing for 30 s and incubation in an oven at 50°C for 30 min. Then, 130 µl pyridine (Merck KGaA, Germany) was added, which was followed by incubation in the oven at 50°C for 30 min. The solution was then filtered using syringe filters (nylon syringe filter, Membrane Solutions, Auburn, WA, USA) with 0.45µm pore size prior to gas chromatography-mass spectrometry (GC-MS) analysis.

### GC-MS Analysis

GC-MS analysis was performed as previously described (34, 35) using a QP2010 gas chromatography-mass spectrometer (GC-2010 coupled with a GC-MS QP-2010 Ultra) equipped with an autosampler (AOC-20i+s) from Shimadzu (Tokyo, Japan), using Rtx-5 ms column (30 m length × 0.25 mm inner diameter × 0.25 µm film thickness: Restek, Bellefonte, PA, USA). Helium (99.9% purity) was used as the carrier gas with a column flow rate of 1 ml/min. The column temperature regime was initially adjusted at 35°C for 2 min, followed by an increase in the rate of 10°C/min to reach 250°C. The temperature was then increased by 20°C/min until reaching 320°C and kept for 23 min. The injection volume was 1 µl and the injection temperature was 250°C using splitless injection mode. The mass spectrometer was operated in electron compact mode with electron energy of 70 eV. Both the ion source temperature and the interface temperature were set at 240°C and 250°C, respectively. The MS mode was set on scan mode starting from 35 to 450 *m*/*z* with a scan speed of 1,428. Data collection and analysis were performed using MSD Enhanced Chemstation software (Shimadzu). Product spectra were identified by comparison of the measured fragmentation patterns to those found in the NIST 08 Mass Spectral Library.

### Metabolic Pathways and Functional Enrichment Analysis

The identified unique metabolites in each group of healthy pulp tissue, periapical abscess, radicular cyst, and periapical granuloma were searched in the PubChem database (https://pubchem.ncbi.nlm.nih.gov/) to identify the active genes related to the unique metabolites. These active genes were then annotated and analyzed using the Metascape tool (36) for gene annotation and analysis. The functional enrichment pathways, molecular complex detection (MCODE), and the related protein–protein interaction (PPI) (36) were recorded. The annotated metabolites were subjected to unsupervised hierarchical clustering using Euclidean distance measure and Ward linkage analysis.

### Genome-Wide Association and Related Oral Diseases

The Genome-Wide Association Studies (GWAS) Catalog (https://www.ebi.ac.uk/gwas/) provides a useful approach to look for specific genetic variations or genetic markers for the prediction of a disease (37). In our study, the GWAS Catalog was searched for different oral lesions such as dental caries, periodontitis, oral cancer, and oral ulcers, in addition to periapical abscess, radicular cyst, and periapical granuloma. Within GWAS, the genes that are related to the aforementioned oral diseases were identified. These genes and their single nucleotide polymorphisms (SNPs) are recorded in **Table S1**. Some of these mapped genes in addition to others from published literature were used to validate our data in gene expression analyses.

### Quantitative Real-Time PCR

Clinical samples were used to quantify the gene expression related to functional metabolic pathways. Sample tissues were homogenized and RNAs were extracted using RNeasy Mini Kit (Qiagen, Germany), then reverse transcribed into cDNA using SuperScript™ III first-strand synthesis system (Thermo Fisher Scientific) according to the instructions of the manufacturer. The sequences of employed primers are described in **Table S2**. RT-PCR setup and cycling procedures were done as previously described (38) using QuantStudio RT-PCR (Applied Biosystems, Waltham, MA, USA). 18S rRNA was used for normalization to other target genes and the relative fold change was calculated using 2−^(ΔΔCt)^.

### CIBERSORT Analysis of Immune Cell Infiltrates in the Periapical Area

The active genes of unique metabolites that were identified and retrieved from PubChem were analyzed as groups using *in silico* CIBERSORT tool available at https://cibersort.stanford.edu/. *In silico* CIBERSORT analysis was performed to identify the immune cells participating in each lesion in comparison with healthy control pulp tissue. The reference gene list for the immune cells (39) was downloaded from https://github.com/holiday01/deconvolution-to-estimate-immune-cell-subsets.

### Database Single Nucleotide Polymorphism

In PubChem, all the identified active genes related to the unique metabolites of periapical abscess, radicular cyst, and periapical granuloma were mapped within the Allele Frequency Aggregator (ALFA) in the database single nucleotide polymorphism (dbSNP) using https://www.ncbi.nlm.nih.gov/snp/. All the information regarding chromosome number, position, allele polymorphism, and population with the highest percentage of the alternative alleles in each gene are recorded in **Table 2**.

**Table 2:** The highest distribution of single nucleotide polymorphism in a population of gene-related enrichment pathways of periapical lesions in human genome assembly (GRCh38.p12).

Gene	Accession number	Chromosome	Position	Reference SNP	Alleles	Population	Alternative allele
Periapical abscess							
*MAPT*	NM_001123066.3	Chr. 17	46028029	rs7521	A>G/A>C	East Asian	G = 0.786, C = 0.093
*CYP1A2*	NM_000761.5	Chr. 15	74749576	rs762551	C>A	Other Asian	A = 0.73
*CYP4F2*	NM_001082.5	Chr. 19	15879621	rs2108622	C>T	South Asian	T = 0.3638
*CYP2C9*	NM_000771.4	Chr. 10	94981296	rs1057910	A>C	South Asian	C = 0.1147
*KAT2A*	NM_001376227.1	Chr. 17	42114374	rs9303326	A>G	African Others	G = 0.030
*HSD17B10*	NM_001037811.2	Chr. X	53432086	rs28935475	–	–	–
Radicular cyst							
*POLB*	NM_002690.3	Chr. 8	42361530	rs2272615	A>G	Other African	G = 0.831
*PRKAG2*	NM_001040633.1	Chr. 7	151556140	rs7429	C>T	Other Asian	T = 0.79
*PRKAB2*	NM_005399.5	Chr. 1	147155343	rs6937	T>C	East Asian	C = 0.6641
*F2*	NM_000506.5	Chr. 11	46723453	rs5896	C>T	East Asian	T = 0.5986
*PRKAG3*	NM_017431.3	Chr. 2	218831762	rs60578863	G>A	African Others	A = 0.090
*PRKAA2*	NM_006252.4	Chr. 1	56706197	rs17848599	T>C	Other Asian	C = 0.06
*DPP4*	NM_001379604.1	Chr. 2	162073412	rs17575	C>T	African Others	T = 0.053
*PRKAG1*	NM_001206709.2	Chr. 12	49019703	rs7975791	C>T	European	T = 0.036586
*EHMT2*	NM_001289413.1	Chr. 6	31883845	rs61745377	C>T	African Others	T = 0.033
*PRKAA1*	NM_001355028.2	Chr. 5	40765115	rs56138995	T>C	South Asian	C = 0.01
*GSK3B*	NM_001146156.2	Chr. 3	119923394	rs17183890	C>T	African American	T = 0.0065
*PRKAB1*	NM_006253.5	Chr. 12	119672396	rs115821895	G>A	Latin American 1	A = 0.006
Periapical granuloma							
*PPARD*	NM_001171818.2	Chr. 6	35428018	rs9794	G>C	Other African	C = 1.0
*TSHR*	NM_000369.5	Chr. 14	81144239	rs1991517	G>C	Other African	C = 0.943
*SLC16A1*	NM_001166496.1	Chr. 1	112912477	rs7169	G>A	Other African	A = 0.907
*TERT*	NM_001193376.2	Chr. 5	1266195	rs2075786	A>G	European	G = 0.64999
*AR*	NM_000044.6	Chr. X	67545785	rs6152	G>A	African Others	A = 0.631
*HCAR1*	NM_032554.4	Chr. 12	122731922	rs496911	G>A	East Asian	A = 0.5804
*GPR84*	NM_020370.3	Chr. 12	54365598	rs2370872	A>G	Latin American 1	G = 0.541
*ESR1*	NM_000125.4	Chr. 6	152122254	rs12681	C>T	African Others	T = 0.275
*NR0B1*	NM_000475.5	Chr. X	30309250	rs6150	G>A	European	A = 0.20578
*PPARA*	NM_001001928.3	Chr. 22	46218377	rs1800206	C>G	European	G = 0.06122
*SLC22A8*	NM_001184732.1	Chr. 11	62993276	rs11568487	G>T	African Others	T = 0.028
*SLC22A6*	NM_004790.5	Chr. 11	62977279	rs11568635	G>A	African American	A = 0.0120
*ESRRA*	NM_001282450.1	Chr. 11	64307271	rs117285599	C>T	Latin American 1	T = 0.011
*RXRA*	NM_001291920.1	Chr. 9	134401726	rs1805337	C>T	Asian	T = 0.002
*PPARG*	NM_001330615.4	Chr. 3	12381349	rs1800571	–	–	–

### Statistical Analysis

The metabolomic data including metabolite names and the corresponding area under the peak values were transferred from the GC-MS to an Excel worksheet, followed by filtration and organization. Area under the peak values for all metabolites were exported to R programming language (version 3.6.2) to generate Venn diagram and heatmap for metabolite distribution (**Tables S3, S4**). GraphPad Prism software (version 9.1.0) was used to generate the column graphs for metabolite group comparison and gene expression. Quantitative comparisons of shared and unique metabolites were analyzed by two-way analysis of variance (ANOVA) and one-way ANOVA, respectively, using Tukey’s multiple comparisons test as indicated per graph. Data of qRT-PCR of the selected genes were analyzed by ANOVA using Tukey’s multiple comparison test. *P*-value ≤0.05 was considered as significant. Fold changes of the weighted average of area under the peak values were calculated to compare the level of metabolites between the lesions and the healthy controls, between lesions only, or within a lesion. One metabolite with a median value among the others was regarded as a reference for calculating the approximate fold value of the other metabolites. For shared metabolites in lesions and healthy pulp control, hexadecane in healthy control was used as a reference representing one-fold. For the shared metabolites between lesions, 2-methylhexacosane in periapical abscess was regarded as a reference representing one-fold. 10-Undecynoic acid, 3-ethyl-5-(2-ethylbutyl)-octadecane, ethanimidic acid, and octanoic acid were employed as one-fold to calculate the fold change of the corresponding metabolites in their respective unique group in healthy control, periapical abscess, radicular cyst, and periapical granuloma, respectively.

### Study Approval

Ethical approval for this study was sought and granted by the Research Ethics Committee at the University of Sharjah (Reference number REC-18-12-17-02-S on February 17, 2019). Informed consent was obtained from all patients who participated in the study. Samples were collected from the Oral and Maxillofacial Department and from the Emergency Dental Care Department of the Medical Dental College at the University of Sharjah.

## Results

**Figure 1:**
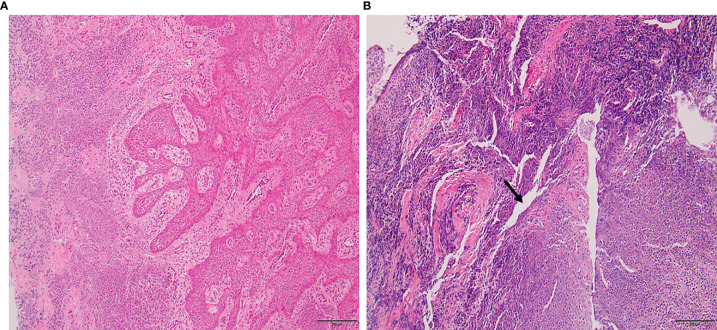
Histopathology of periapical lesions. **(A)** Periapical granuloma with well-organized epithelium and connective tissue infiltrated with inflammatory cells. **(B)** Radicular cyst with epithelialized lumen and chaotic arrangement of cells. An arrow indicated the lipid cleft in radicular cyst. Tissues were stained with hematoxylin and eosin with ×10 magnification power.

**Figure 2:**
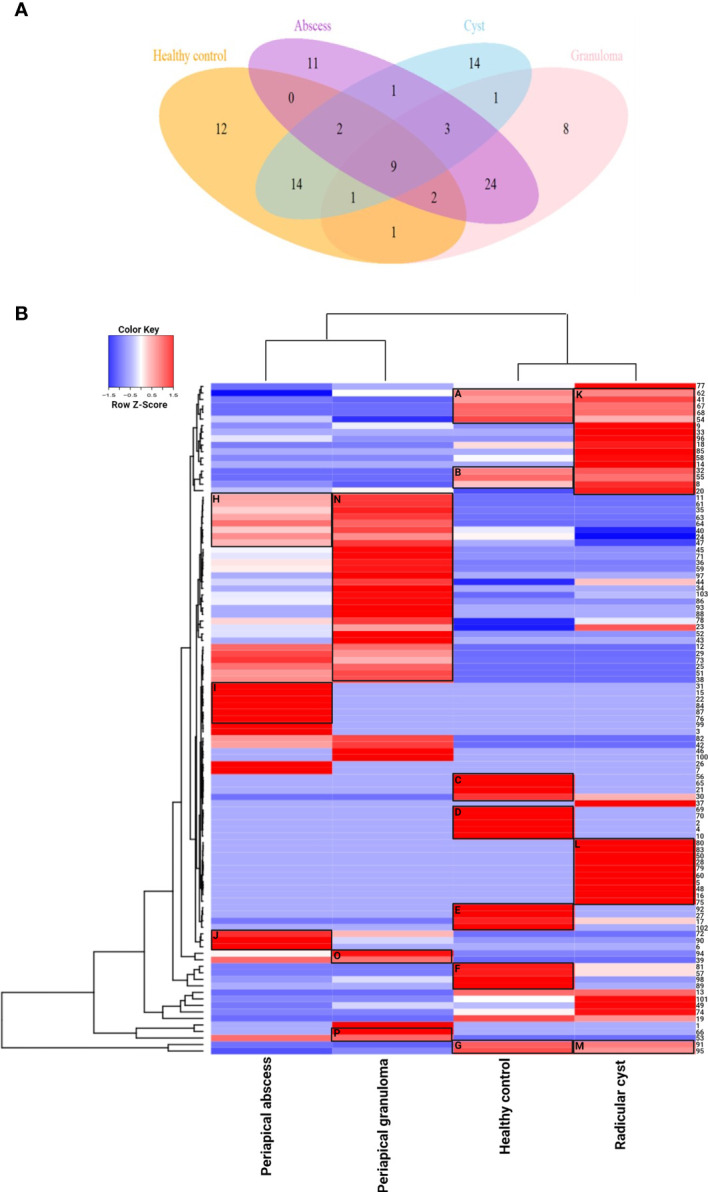
Metabolite distribution in periapical lesions and healthy pulp tissues. **(A)** Venn diagram representing the number of unique and shared metabolites in periapical abscess, radicular cyst, periapical granuloma, and healthy control. The color code is the same as that of **Table 3** which lists the most important metabolites in full. The unique metabolites in each group were 14, 12, 11, and 8 metabolites in radicular cysts, healthy control, periapical abscesses, and periapical granulomas, respectively. The shared metabolites were as follows: 24 metabolites in periapical abscesses and granulomas; 14 metabolites in healthy control and radicular cysts; 9 metabolites in all groups; 3 metabolites in all periapical lesion groups; 2 metabolites in healthy control, periapical abscesses, and radicular cysts; and 2 metabolites in healthy control, periapical abscesses, and periapical granulomas. Only one metabolite was found to be shared between periapical abscesses and radicular cysts; between radicular cysts and periapical granulomas; between healthy control and periapical granulomas; and between healthy control, radicular cysts, and periapical granulomas. **(B)** Unsupervised hierarchical clustering and heatmap analysis of the identified metabolites in the periapical lesions and healthy control. The values of metabolites represented the weighted average of area under the peak in each group of healthy control, periapical abscess, radicular cyst, and periapical granuloma. Euclidean distance measure and Ward linkage analysis were used to carry out unsupervised hierarchical clustering using the metabolomics data. Heatmap analysis showed that the metabolites clustered into two separate groups, a group representing the healthy control and radicular cyst and the other group representing the periapical abscess and granuloma. In the healthy control group, carboxylic acid metabolites in cluster C are included in dental pellet composition, while cluster D metabolites are responsible for normal signal transduction and antimicrobial activity. Cluster E metabolites enhance NK cells with some antifungal activities. Oleic acid in cluster F has anti-inflammatory and antimicrobial activities, in addition to dendritic cell activation. Cluster G metabolites are known to activate NK cells and dendritic cells and induce apoptotic cellular response. In the periapical abscess, cluster H metabolites are correlated to M2 polarization and IL-10 expression. Cluster I metabolites activate NK cell; increase MMP-9, IL-6, and IL-10 release; and downregulate M1 macrophage polarization. Cluster J metabolites have a pro-inflammatory activity and activate the production of IL-8, CYP4F3, and VEGF. In the radicular cyst, cluster K metabolites are generally related to cellular membrane flexibility and radicular cyst expansion. Cluster L metabolites are highly hydrophobic lipid metabolites released during lipophagy. Cluster M metabolites are significantly correlated to induce apoptosis. In the periapical granuloma, cluster N metabolites induce anti-inflammatory M2 polarization. Cluster O metabolites maintain the chronic inflammation and induce M2 macrophage polarization. Cluster P contains L-(+)-lactic acid and ethylene glycol that appear to be involved in granuloma formation, polarize more M2 macrophage, and reduce the cytotoxic effect of NK cells.

The lesion samples used in this study included periapical abscesses, radicular cysts, and periapical granulomas. Both radicular cysts and periapical granulomas were discriminated following histopathological examination (**Figure 1**). The metabolic profiling of both healthy pulp tissue (used as a control) and lesion samples was performed using GC-MS analysis. A flowchart summarizes the methodological procedures in **Figure S1**. The identified metabolites were compared, classified, and correlated with the clinical characteristics of the investigated lesion types. Over a hundred metabolites were identified, some of them were shared between lesions and the healthy control, others were shared between the lesions only, and others were unique to a lesion type (**Figure 2A**, **Table 3**, and **Table S5**). Heatmap analysis showed that the identified metabolites clustered into two separate groups: a group representing the healthy control and radicular cyst and the other group representing the periapical abscess and granuloma (**Figure 2B** and **Tables S3**, **S4**). Unique metabolites to a lesion type in comparison with healthy pulp tissue were mapped as shown in **Table 3** and **Table S5**. Metabolite occurrence was classified as below.

**Table 3:** Occurrence of metabolites in periapical lesions compared with healthy control.

Metabolites^a^	Cluster	PubChem ID	Healthy control	Periapical abscess	Radicular cyst	Periapical granuloma
Palmitic acid	GM	985	√	√	√	√
Stearic acid	GM	5281	√	√	√	√
Eicosane	_	8222	√	√	√	√
2-Bromotetradecane	K	12798926	√	√	√	√
Hexadecane	AK	11006	√	√	√	√
Citric acid	AK	311	√	√	√	√
Myristic acid	_	11005	√	√	√	√
2,6,11-Trimethyl dodecane	HN	35768	√	√	√	√
Urea	N	1176	√	√	√	√
Nonadecane	N	12401	0	√	√	√
2-Methylhexacosane	N	150931	0	√	√	√
Disulfide, di-tert-dodecyl	N	117981	0	√	√	√
Oleic acid	F	445639	√	0	0	0
1,54-Dibromo-tetrapentacontane	E	545963	√	0	0	0
Pentadecanoic acid	E	13849	√	0	0	0
3-Dodecanol	E	139108	√	0	0	0
2-Hexadecyl-1-eicosanol	D	86602	√	0	0	0
10-Undecynoic acid	D	31039	√	0	0	0
L-Aspartic acid	D	5960	√	0	0	0
L-Glutamic acid	D	33032	√	0	0	0
2-Methyl-1,5-heptadiene-3,4-diol	D	5366259	√	0	0	0
2-Butenedioic acid	C	444972	√	0	0	0
Itaconic acid	C	811	√	0	0	0
Glycolic acid	C	757	√	0	0	0
17-Octadecynoic acid	J	1449	0	√	0	0
4,4-Dimethoxy-2-methyl-2-butanol	I	4439980	0	√	0	0
2,2-Dimethyl-1-octanol	I	520068	0	√	0	0
2-Hydroxy-3-methylbutyric acid	I	99823	0	√	0	0
Octacosane	I	12408	0	√	0	0
3-Ethyl-5-(2-ethylbutyl)-octadecane	I	292285	0	√	0	0
Methyl isovalerate	I	11160	0	√	0	0
1-Iodo-tetracosane	_	11282694	0	√	0	0
10-Methylnonadecane	_	530070	0	√	0	0
2-Propenoic acid	_	6581	0	√	0	0
2,2-Dimethyl-1-decanol	_	520069	0	√	0	0
1-Nonadecene	K	29075	0	0	√	0
5,5-Diethylpentadecane	K	85977274	0	0	√	0
Octadecane	K	11635	0	0	√	0
Methoxyacetic acid 4-hexadecyl ester	L	545959	0	0	√	0
5-Butyl-nonane	L	300476	0	0	√	0
Nonyl tetracosyl ether	L	87077463	0	0	√	0
Ethanimidic acid	L	178	0	0	√	0
8-Methyl-heptadecane	L	292723	0	0	√	0
3-Ethyl-3-methylheptane	L	140213	0	0	√	0
Nonadecyl pentafluoropropionate	L	91693316	0	0	√	0
11-Methyldodecanol	L	33865	0	0	√	0
Dodecyl nonyl ether	L	87077689	0	0	√	0
1-Piperidinecarboxaldehyde	L	17429	0	0	√	0
Beta-Sitosterol	_	222284	0	0	√	0
L-(+)-Lactic acid	P	107689	0	0	0	√
Decanoic acid	N	2969	0	0	0	√
Petroselinic acid	N	5281125	0	0	0	√
Octanoic acid	N	379	0	0	0	√
6-Ethyl-3-decanol	N	140584	0	0	0	√
Sulfurous acid, 2-propyl tridecyl ester	N	6420355	0	0	0	√
2,6,10-Trimethyl-dodecane	_	19773	0	0	0	√
2,6,10-Trimethyl-tetradecane	_	85785	0	0	0	√

a The colored metabolites are linked to **Figure 2A** color code.

### Metabolite Clustering Reveals Differences Between Lesions and Healthy Pulp Tissues

In the healthy control group, clusters A, B, and G showed clear a overlap with clusters K and M in radicular cyst, while no overlap with periapical abscess or granuloma was observed. Unique metabolites to healthy pulp tissue were clustered in C, D, E, and F as follows: cluster C contains glycolic acid, itaconic acid, and 2-butenedioic acid. Cluster D contains L-aspartic acid, L-glutamic acid, and 10-undecynoic acid. While cluster E contains pentadecanoic acid and 3-dodecanol, cluster F contains oleic acid. Cluster G contains palmitic and stearic acids, which are shared with radicular cysts but in lower concentrations (**Figure 2B** and **Table S3**).

In the periapical abscess, metabolites in cluster H overlapped with the first part of cluster N of the periapical granuloma, but with different concentrations. For example, arsenous acid and cholesterol are present in lower concentrations in periapical abscess than in periapical granuloma. Cluster I contains unique metabolites including 4,4-dimethoxy-2-methyl-2-butanol, 2-hydroxy-3-methylbutyric acid, 3-ethyl-5-(2-ethylbutyl)-octadecane, and methyl isovalerate. Cluster J contains linoelaidic acid, oxalic acid, and 17-octadecynoic (**Figure 2B** and **Table S3**).

In the radicular cyst, metabolites in cluster K are alkanes and alkene such as hexadecane, 5,5-diethylpentadecane, octadecane, heptadecane, 1-nonadecene, 5,5-diethylheptadecane, and 2-bromotetradecane. Interestingly, cluster L appears to delineate the radicular cyst from healthy control and other periapical lesions. Cluster L contains nonane alkanes and their derivatives including 5-butyl-nonane, nonyl tetracosyl ether, nonadecyl pentafluoropropionate, and dodecyl nonyl ether, while cluster M metabolites included saturated fatty acids such as palmitic and stearic acids (**Figure 2B** and **Table S3**).

In the periapical granuloma, cluster N contains arsenous acid, cholesterol, octanoic acid, L-serine, ethanolamine, and butanoic acid 2-methyl-3-oxo- ethyl ester. Cluster O metabolites included phosphoric acid and butylated hydroxytoluene. Cluster P contains L-(+)-lactic acid and ethylene glycol (**Figure 2B** and **Table S3**).

### Quantitative Differences of Metabolites in Lesions and Healthy Pulp Tissues

**Figure 3:**
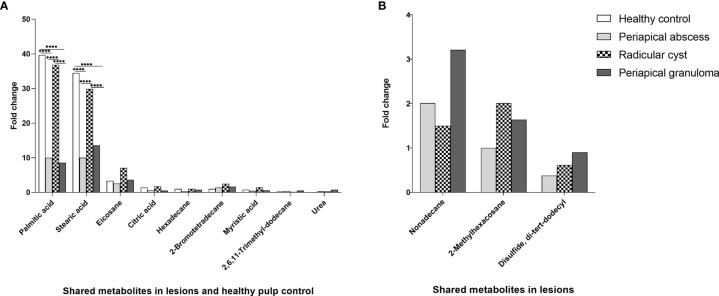
Quantitative comparison of shared metabolites in periapical lesions and healthy control. The quantity of each metabolite was represented as the area under the peak of each metabolite in relation to the total areas of all other metabolites detected in an extract. The data displayed the fold change of area under the peak values of each metabolite in each group. The data were analyzed by two-way analysis of variance (ANOVA) using Tukey’s multiple comparisons test. *P*-value ≤ 0.05 was considered as significant. **(A)** Shared metabolites between periapical lesions and healthy control. Hexadecane in healthy control was regarded as one-fold and employed as a reference for the measurement of the other metabolites. **(B)** Shared metabolites between periapical lesions only. 2-Methylhexacosane in periapical abscess was used as a reference of one-fold for the measurement of metabolites in other lesions. **** reveals that *P*-value < 0.0001.

Palmitic acid, stearic acid, eicosane, citric acid, hexadecane, 2-bromotetradecane, myristic acid, 2,6,11-trimethyl-dodecane, and urea were among the metabolites detected in all lesion types and healthy pulp tissue (**Figure 3A**, **Table 3**, and **Table S5**). Palmitic and stearic acids were the most abundant metabolites in healthy control and lesions in comparison with other metabolites (**Figure 3A**). Palmitic acid and stearic acid were significantly high in healthy control and radicular cyst in comparison with periapical abscess and granuloma (*P *< 0.0001). Other metabolites did not show significant differences between healthy control and lesions (**Figure 3A**). On the other hand, metabolites including nonadecane, 2-methylhexacosane, and disulfide di-tert-dodecyl were detected in lesions (periapical abscess, radicular cyst, and periapical granuloma) but without significant differences (**Figure 3B**, **Table 3**, and **Table S5**).

### Metabolites Specific to Periapical Lesions and Healthy Pulp Tissues

**Figure 4:**
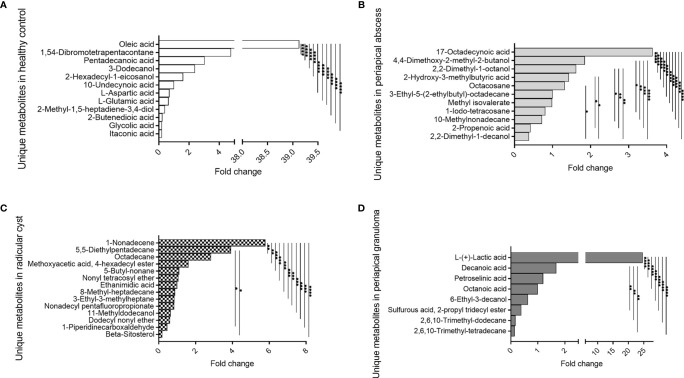
Unique metabolites exclusively identified in specific lesion type compared with healthy control. 10-Undecynoic acid value was regarded as one-fold change and employed for the measurement of other metabolites in healthy pulp control. 3-Ethyl-5-(2-ethylbutyl)-octadecane value was regarded as one-fold change and employed for the measurement of other metabolites in periapical abscess. Ethanimidic acid value was regarded as one-fold change and employed for the measurement of other metabolites in radicular cyst, and octanoic acid value was regarded as one-fold and employed as a reference for the measurement of fold change for the other metabolites in periapical granuloma. The data display the fold change of the area under the peak of unique metabolites in each group. The data were analyzed using one-way analysis of variance (ANOVA) using Tukey’s multiple comparisons test. *P*-value ≤0.05 was considered as significant. **(A)** Metabolites identified only in healthy control. **(B)** Metabolites identified only in periapical abscess. **(C)** Metabolites identified only in periapical cyst. **(D)** Metabolites identified only in periapical granuloma. * reveals that *P*-value < 0.05, ** reveals that *P*-value < 0.01, *** reveals that *P*-value < 0.001, **** reveals that *P*-value < 0.0001.

Several metabolites were exclusively identified in specific lesions in comparison with the healthy control group (**Figure 4**, **Table 3**, and **Table S5**). Metabolites identified in healthy control pulp tissues included oleic acid, 1,54-dibromotetrapentacontane, pentadecanoic acid, 3-dodecanol, 2-hexadecyl-1-eicosanol, 10-undecynoic acid, L-aspartic acid, L-glutamic acid, 2-methyl-1,5-heptadiene-3,4-diol, 2-butenedioic acid, glycolic acid, and itaconic acid. Oleic acid was significantly high in healthy control in comparison with other metabolites (*P *< 0.0001) (**Figure 4A**, **Table 3**, and **Table S5**). In periapical abscess, 17-octadecynoic acid, 4,4-dimethoxy-2-methyl-2-butanol, 2,2-dimethyl-1-octanol, 2-hydroxy-3-methylbutyric acid, octacosane, 3-ethyl-5-(2-ethylbutyl)-octadecane, methyl isovalerate, 1-iodo-tetracosane, 10-methylnonadecane, 2-propenoic acid, and 2,2-dimethyl-1-decanol were identified. 17-Octadecynoic acid was the most abundant metabolite significantly identified in periapical abscess in comparison with all other metabolites (*P *< 0.0001) (**Figure 4B**, **Table 3**, and **Table S5**). In radicular cyst, 1-nonadecene, 5-5-diethylpentadecane, octadecane, methoxyacetic acid 4-hexadecyl ester, 5-butyl-nonane, nonyl tetracosyl ether, ethanimidic acid, 8-methyl-heptadecane, 3-ethyl-3-methylheptane, nonadecyl pentafluoropropionate, 11-methyldodecanol, dodecyl nonyl ether, 1-piperidinecarboxaldehyde, and beta-sitosterol were identified. 1-Nonadecene was the most abundant metabolite significantly identified in radicular cyst in comparison with beta-sitosterol (*P *< 0.0001), 1-piperidinecarboxaldehyde, dodecyl nonyl ether, 11-methyldodecanol, nonadecyl pentafluoropropionate, 3-ethyl-3-methylheptane, 8-methyl-heptadecane, ethanimidic acid, nonyl tetracosyl ether, 5-butyl-nonane, methoxyacetic acid 4-hexadecyl ester (*P *< 0.001), octadecane, and 5-5-diethylpentadecane (*P *< 0.01) (**Figure 4C**, **Table 3**, and **Table S5**). Metabolites including L-(+)-lactic acid, decanoic acid, petroselinic acid, octanoic acid, 6-ethyl-3-decanol, sulfurous acid, 2-propyl tridecyl ester, 2,6,10-trimethyl-dodecane, and 2,6,10-trimethyl-tetradecane were only identified in periapical granuloma. L-(+)-lactic acid was significantly the most detectable metabolite in comparison with the aforementioned metabolites in periapical granuloma (*P *< 0.0001) (**Figure 4D**, **Table 3**, and **Table S5**).

### Metabolic Pathways and Functional Enrichment Analysis Separated the Lesions From Healthy Control

**Figure 5:**
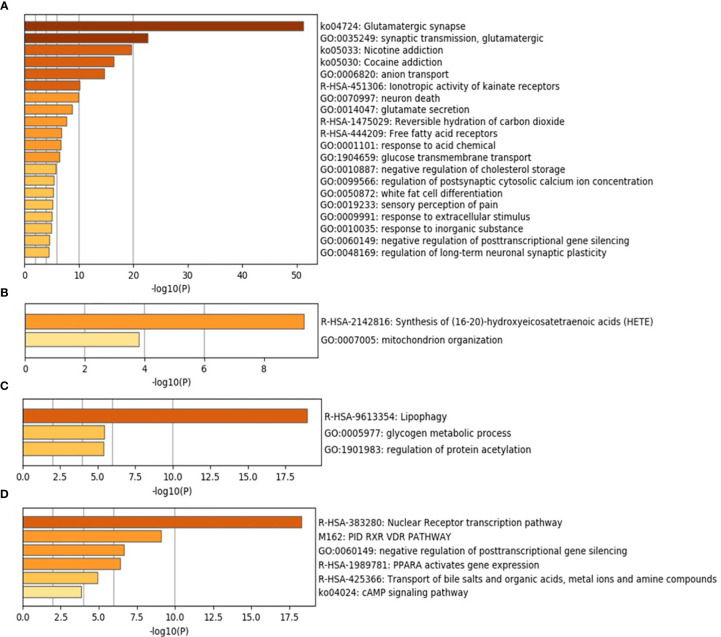
Top 20 pathways and functional enrichment analysis using Metascape. The *y*-axis shows significantly enriched gene ontology terms of the target genes. The darker the color, the higher the gene counts included in that pathway. The *x*-axis shows the enrichment scores of the gene ontology terms. *P*-value is calculated according to the count of the provided genes found in the given pathway. Log10(*P*) is the *P*-value in log base 10. *P*-value ≤0.05 was considered as significant. **(A)** Healthy control. **(B)** Periapical abscess. **(C)** Radicular cyst. **(D)** Periapical granuloma.

Metascape was used to predict the different enrichment pathways grouped for each lesion compared with healthy tissues (**Figure 5** and **Figure S1**). The relation between the top enriched pathways in each group and their corresponding gene expression is described in **Table 4**. Considerably, a high number of pathways were found in healthy controls representing homeostatic conditions (**Table 4** and **Figure 5A**). The MCODE algorithm revealed the functional description of the corresponding gene ontology with PPI. The highest MCODE-1 was for glutamate binding and activation followed by MCODE-2 for G alpha (q) signaling events and MCODE-3 for ionotropic activity of kainate receptors (**Table S6**). PPI in healthy control was highly observed between GRIN-GRIA proteins followed by FFAR-GRM proteins and lastly between GRIK-G proteins (**Figure S2A**). In the periapical abscess, the synthesis of 16-20-HETE and the mitochondrion organization pathways were the most prevalent (**Table 4** and **Figure 5B**). Only MCODE-1 with the highest significance for the synthesis of 16-20-HETE (**Table S6**) and the highest PPI in periapical abscess was between cytochrome P450 proteins (**Figure S2B**). Lipophagy, glycogen metabolic process, and regulation of protein acetylation pathways were the highest abundant pathways in cystic tissues (**Table 4** and **Figure 5C**). MCODE-1 with the highest significance for the activation of AMPK (**Table S6**) and the highest PPI in radicular cyst was between PRKA proteins (**Figure S2C**). Nuclear receptor transcription factors (RXR and VDR), posttranscriptional gene silencing, and PPARA pathways were the most abundant pathways related to periapical granuloma (**Table 4** and **Figure 5D**). MCODE-1 with the highest significance for nuclear receptor transcription pathway (**Table S6**) and the highest PPI in periapical granuloma was between RXRA, PPARG, ESR1, ESRRA, and NR0B1 proteins (**Figure S2D**).

**Table 4:** The relation between the unique metabolites, enrichment pathways, and the related functional genes in periapical lesions and healthy controls.

Group	Unique metabolites	Enrichment pathway	Associated genes to the enriched metabolic pathway and tissue condition
Periapical abscess	•17-Octadecynoic acid	•R-HSA-2142816: synthesis of (16–20)-hydroxyeicosatetraenoic acids (HETE) •GO:0007005: mitochondrion organization	•The amount of total lipid increased during abscess development (40). 20-HETE is a lipid eicosanoid metabolite derived from the metabolism of arachidonic acid (41). Human PMNs express CYP4F3 (42), which produces 20-HETE (43). HETE potentiates angiogenesis, inflammation, and apoptosis (44, 45). HETE potentiates VEGF expression (46) and MMP-9 (47) but compromises the survival and function of endothelial cells for collateral vessel formation (48), thus interfering with blood vessel formation. HETE increases reactive oxygen species production and NF-κB activity. This results in endothelial activation characterized by the increase in the expression of IL-8 (49). Bacteroides surface protein A (BspA) is identified in oral bacteria like *Tannerella forsythia* (50), the major oral pathogens in abscess formation (51). Binding of BspA to TLR2 causes the release of chemokine IL-8 from the human gingival epithelial cells (52). •Abscess in general is an osmotically active environment (53) and mitochondrial remodeling occurs during hyperosmotic stress (54). •In conclusion, upregulation of CYP4F3 in periapical abscess enhances the production of 20-HETE, which in turn upregulated VEGF and MMP-9 and activated NF-κB and hence increased the expression of IL-8. Furthermore, upregulation of TLR2 has a stimulatory effect on the upregulation of IL-8.
Radicular cyst	•Beta-sitosterol •Ethanimidic acid	•R-HSA-9613354: lipophagy •GO:0005977: glycogen metabolic process •GO:1901983: regulation of protein acetylation	•Lipophagy is used to describe the autophagic degradation of lipid droplets (55). Nutrient depletion promotes lipophagy (56, 57). Nutritional deficiency is the most proposed theory for the formation of radicular cyst (58). Lipophagy was reported in lipid-laden macrophages (foam macrophages) (59, 60) and in memory CD8 T cells (61) in odontogenic cyst (62–64). IL-12 is important in CD8 T-cell clonal expansion in addition to the generation of memory CD8 T cells (65). IL-12 released from macrophages inhibits VEGF and MMP-9 (66). IL-17A released from Th-17 enhances either directly bone resorption in periapical lesions (67) or indirectly through stimulating autophagy in macrophages and osteoclast differentiation (68). •High cellular metabolism in radicular cyst is due to its inflammatory origin (69) and glycogenolysis is necessary for inflammatory macrophage survival (70). •Histone acetyltransferase p300 showed significant higher expression in periapical cyst in comparison with healthy tissue (71). •In conclusion, IL-12A was the most prominent upregulated gene, which is highly correlated to CD8 effector and memory CD8 T cells, which were reported in our study to be the highest lymphocytes present in radicular cyst (**Figure 7** and **Table S7**) with enhanced lipophagy. Additionally, IL-12 has an inhibitory effect on the expression of VEGF and MMP-9. IL-17, which is related to bone resorption in periapical lesions, also stimulates the autophagy in macrophages and osteoclasts.
Periapical granuloma	•L-(+)-Lactic acid •Octanoic acid •Decanoic acid •Petroselinic acid	•R-HSA-383280: nuclear receptor transcription pathway M162: PID RXR VDR PATHWAY •GO:0060149: negative regulation of posttranscriptional gene silencing •R-HSA-1989781: PPARA activates gene expression	•Retinoid X receptor (RXR) has sequence similarities to ROR subfamily of NRs (72). Retinoic acid-related orphan receptor gamma t (RORγt) is a nuclear receptor, which is selectively expressed by various lymphocytes. RORγt is critical for the development of secondary and tertiary lymphoid organs and for the thymic development of T-cell lineage (73). RORγt has been extensively studied as the master transcription factor of IL-17 expression and Th17 cells, which are strongly associated with various inflammatory and autoimmune conditions (73). Positive correlations between RORγt and IL-17 protein levels were observed in periapical granulomas (74). Vitamin D receptor (VDR) is an endocrine member of the nuclear receptor superfamily (75). The 1α,25-(OH)2 D3/VDR complex functions to regulate gene transcription through heterodimerization with any of three retinoid X receptor (RXR) isoforms and binds to cognate vitamin D responsive elements (VDREs) in the promoter region of target genes (76). This explains the massive local production of 1,25-(OH)2D by disease-associated macrophages that is seen in patients with granulomatous diseases (77, 78) and even granulation tissue formation in normal wound healing (79). Abnormality in VDR gene related to apical periodontitis (80). Activation of VDR can downregulate MMP-9 in vascular cells (81). •Methylation is related to gene silencing (82). Hypomethylation of TLR2 promoter exacerbates periapical inflammatory and angiogenic responses in association with symptomatic apical periodontitis (83). •PPAR-α is activated under conditions of energy deprivation and prolonged starvation (84, 85). This condition can be observed with a high level of lactic acid (86). MMP-9 protein was not affected by PPARα activators (87) but negatively regulated by VDR pathway (81). PPARα negatively regulates TLR4 activity and therefore exerts anti-inflammatory actions (88). PPARα agonists are found to inhibit endothelial VEGFR2 expression (89). •In conclusion, IL-17A was significantly upregulated due to the activation of RORγt in Th17. TLR4 was significantly downregulated in granuloma compared with healthy (*P* *** ***<** **0.05), which was most probably due to the negative regulation of PPARα. Due to the inhibitory effect of PPARα and VDR pathways, VEGFR and MMP-9 were also downregulated, respectively.
Healthy control	•Oleic acid •L-Glutamic acid •L-Aspartic acid •Pentadecanoic acid •2-Butenedioic acid •Itaconic acid •Glycolic acid	•ko04724: glutamatergic synapse •GO:0035249: synaptic transmission, glutamatergic •ko05033: nicotine addiction •ko05030: cocaine addiction •GO:0006820: anion transport •R-HSA-451306: ionotropic activity of kainate receptors •GO:0070997: neuron death	•Vesicular glutamate transporters (VGLUTs) are involved in the transport of transmitter glutamate into synaptic vesicles and are used as markers for glutamatergic neurons. VGLUT1 is involved mainly in the glutamate-mediated signaling of pain, primarily at the level of healthy peripheral dental pulp (90). •Some patients are smokers which explain the inclusion of nicotine-enriched pathway. •Lidocaine used as local anesthetic solution is one of cocaine derivatives (91). •The organic anion transporter family is known to play an important role in the elimination of a variety of endogenous and exogenous harmful substances from the body (92). Tooth enamel formation or amelogenesis is roughly divided into two consecutive stages: the secretory stage and the maturation stage. In the secretory stage, tall columnar ameloblasts synthesize and secrete enamel matrix proteins. Once the full thickness of the enamel is laid down, the ameloblasts become typical transporting cells and regulate calcium influx and matrix removal in and out of the enamel throughout the process of enamel maturation (93, 94). For the highly mineralized enamel to form, extensive degradation and reabsorption of the organic matrix are essential (93). •Kainate receptors are ionotropic glutamate receptors that mediate fast excitatory neurotransmission and are localized to the presynaptic and postsynaptic sides of excitatory synapses (95). Glutamate receptor ionotropic kainate 1 (GRIK1) has been implicated in tooth development and root formation (96). •In neural death, apoptosis is a part of normal pulp homeostasis (97). Most apoptotic cells in normal pulp can be found at the periphery and are usually associated with the subodontoblastic region rather than with the odontoblastic layer (98). •In conclusion, the healthy control is characterized by a pulp with densely innervated and highly vascularized soft tissues, and hence, VGLUT1 is necessary for the continuous homeostasis of healthy dental pulp. Healthy dental pulps were obtained from completely impacted wisdom tooth expecting the final stages of enamel mineralization in which the organic matrix should be replaced by the inorganic one through organic anion transporter mechanism.

### Expression of Unique Genes Were Correlated to the Metabolic Pattern of Each Lesion Compared With Healthy Pulp Tissues

The expression of genes identified from the enriched pathways was analyzed next. *CYP4F3*, *VEGF*, *MMP-9*, *IL-8*, and *TLR2* represented the most upregulated genes in periapical abscess (**Table 4**). *IL-17A* was the highest positively correlated gene to periapical granuloma, while *TLR2*, *TLR4*, *VEGF*, and *MMP-9* were negatively correlated with the enriched pathways of granuloma. *IL-6* (99) and *IL-10* (100) are known to master inflammation (**Table 4**) and *IL-12A* is known to play controversial roles in periapical granuloma and cyst (14) with the highest gene polymorphism related to oral diseases (**Table 4** and **Table S1**).

**Figure 6:**
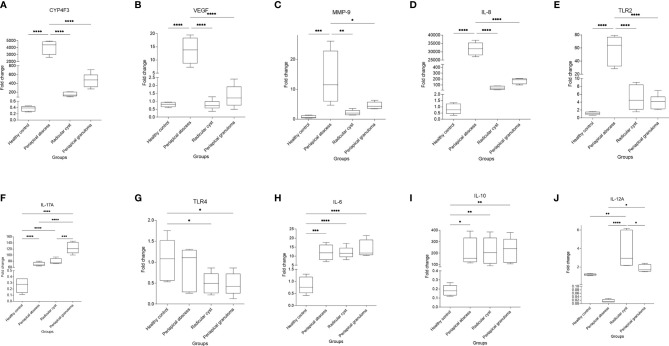
Quantitative real-time PCR of selected genes identified in correlation to enriched pathways in periapical lesions compared with healthy control. Ten clinical samples in each group were investigated and each sample was replicated three times. The data display the fold change in gene expression between groups. The data were analyzed by one-way analysis of variance (ANOVA) using Tukey’s multiple comparisons test. *P*-value ≤0.05 was considered as significant. **(A)** CYP4F3. **(B)** VEGF. **(C)** MMP-9. **(D)** IL-8. **(E)** TLR2. **(F)** IL-17A. **(G)** TLR4. **(H)** IL-6. **(I)** IL-10. **(J)** IL-12A. * reveals that *P*-value < 0.05, ** reveals that *P*-value < 0.01, *** reveals that *P*-value < 0.001, **** reveals that *P*-value < 0.0001.

Significant higher expression of *CYP4F3* (*P *< 0.0001) (**Figure 6A**), *VEGF* (*P *< 0.0001) (**Figure 6B**), *MMP-9* (*P *< 0.001) (**Figure 6C**), *IL-8* (*P *< 0.0001) (**Figure 6D**), and *TLR2* (*P* ******<** **0.0001) (**Figure 6E**) were noted in the periapical abscess compared with healthy controls. The expression of *IL-17A* was significantly upregulated in the periapical granuloma, radicular cyst, and periapical abscess in comparison with healthy controls (*P *< 0.0001) (**Figure 6F**). Interestingly, *TLR4* gene expression was significantly high in healthy pulp tissue compared with the periapical granuloma and cyst (*P *< 0.05) (**Figure 6G**). Furthermore, *IL-6* gene expression was significantly upregulated in the periapical abscess (*P* ******<** **0.001), radicular cyst, and periapical granuloma (*P *< 0.0001) when compared with healthy controls (**Figure 6H**). *IL-10* was significantly high in periapical granuloma, radicular cyst (*P* ******<** **0.01), and periapical abscess (*P *< 0.05) in comparison with healthy controls (**Figure 6I**). *IL-12A* gene expression was significantly increased in the radicular cyst rather than healthy pulp tissue (*P *< 0.01) (**Figure 6J**).

### CIBERSORT Analysis of Immune Cell Infiltrate in the Periapical Area Showed Unique Correlation to Lesion Type

**Figure 7:**
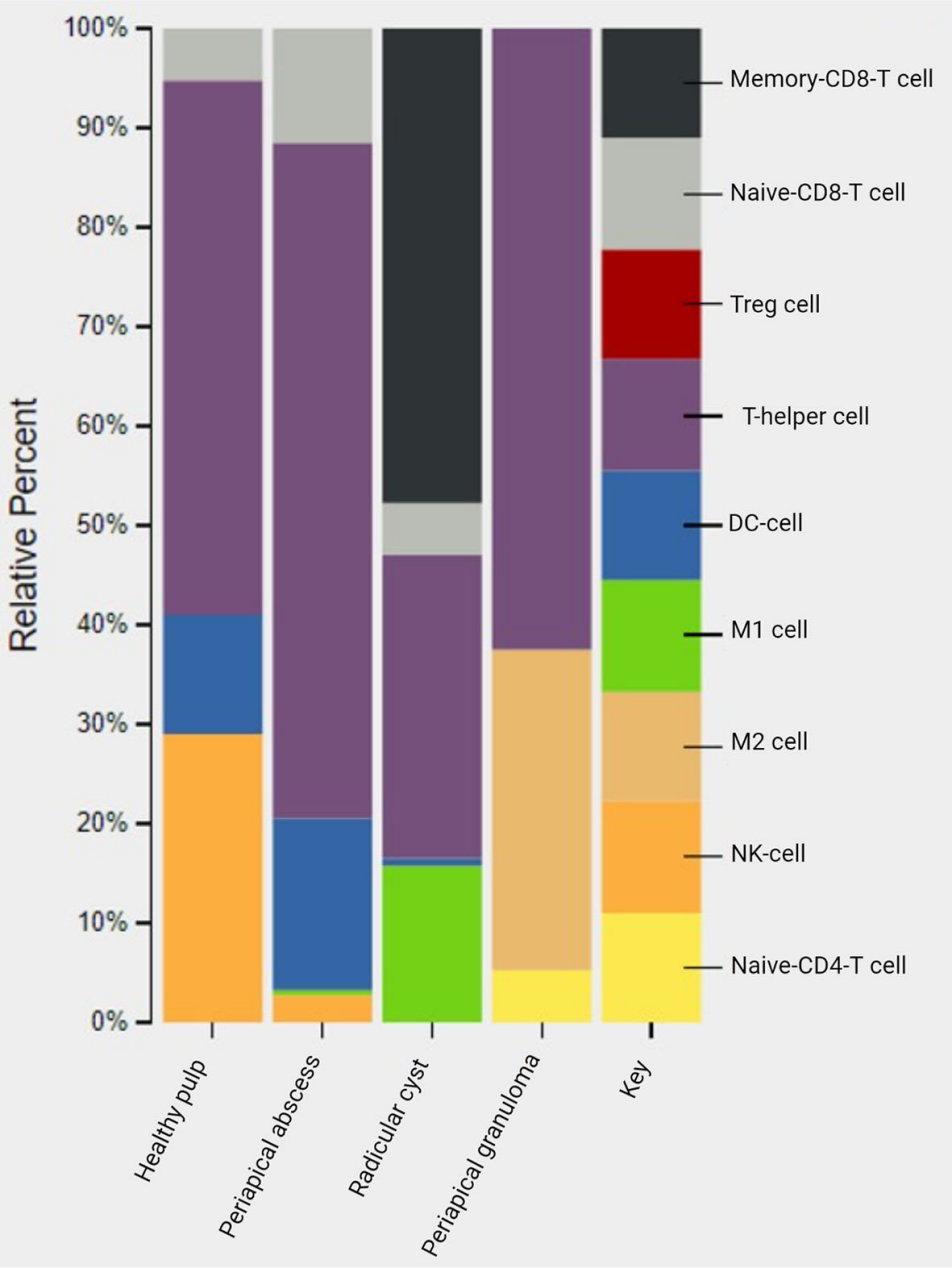
*In silico* deconvolution of immune cells of periapical lesions compared with healthy control using CIBERSORT. The color key represented the reference list of immune cell genes. Treg, T-regulatory cell; DC cell, dendritic cell; M1, pro-inflammatory macrophage; M2, anti-inflammatory macrophage; NK, natural killer cell.

The CIBERSORT system biology tool employs deconvolution of bulk gene expression data and a sophisticated algorithm for *in silico* quantification of many immune cell types in variable samples (101) (**Figure S1**). *In silico* CIBERSORT analysis identified different profiles of immune cells in periapical lesions and healthy control but without significant differences. T-helper cells were found in healthy pulp tissues and in all studied periapical lesions. In healthy pulp tissues, T-helper cells and natural killer (NK) cells were the most common immune cell infiltrates and represented 54% and 29%, respectively. However, a scant presence of dendritic cells and naive CD8 T cells was also found. In periapical abscess, T-helper cells were the most abundant immune cells comprising 68% of the total immune cells, followed by 17% and 11% for dendritic cells and naive CD8 T cells, respectively. The most abundant immune cells in the radicular cysts were the memory CD8 T cells, T-helper cells, and pro-inflammatory M1 macrophages, representing 48%, 31%, and 16% of the total immune cell infiltrates, respectively. Moreover, naive CD8 T cells accounted for only 5% of immune cells in radicular cyst. On the other hand, T-helper cells accounted for 62% and the anti-inflammatory macrophages M2 accounted for 32% in the periapical granuloma (**Figure 7** and **Table S7**).

### Prediction of Pathways Related to SNPs and GWAS Helped Categorize the Periapical Lesions

The common variants within the most related genes of enrichment analysis pathways relevant to the three different lesion types were identified using ALFA. In the periapical abscess, *MAPT* and *CYP1A2* genes had a prediction of alternative alleles of 0.79 and 0.73 from A>G (rs7521) and C>A (rs762551), respectively. The highest polymorphism for *MAPT* was recorded in East Asians, while *CYP1A2* was recorded in other Asian populations (**Table 2**). For radicular cyst, *POLB* and *PRKAG2* genes were identified with A>G (rs2272615) of 0.83 and C>T (rs7429) of 0.79 polymorphism percentage in other African and other Asian population, respectively. In the periapical granuloma, *PPARD* gene was the highest alternative allele prediction from G>C (rs9794) with 1.0 polymorphism percentage in other African populations followed by *TSHR* with a polymorphism percentage of 0.94 from G>C (rs1991517) in the same population (**Table 2**). GWAS for the related oral diseases reveals three oral lesions with their corresponding genes and SNPs. Mouth ulcers were found to be highly correlated to SNPs in *IL-12A* in addition to significant relation to other genes including *IL-10*. In mouth ulcers, several studies reported different SNP positions for the same gene with different significant *P*-values. For example, *IL-19* has three different SNPs in mouth ulcers, while LTA SNP polymorphism is significantly correlated to mouth ulcer. SNPs in LTB are significantly correlated to oral cavity cancer in addition to TNF. On the other hand, dental caries are significantly correlated to SNPs in *IL-17D* (**Table S1**).

## Discussion

Cellular metabolites provide a true image of the interactions between a genome of a specific cell and its environment and, thus, yield an unbiased perspective of the cellular state either in health and disease (102). The metabolomic profiling of periapical lesions and healthy pulp tissue reported here reveals for the first time a metabolite environment that clearly separates the periapical granuloma and abscess from the radicular cyst and healthy control. Indeed, our study sheds light on the role of the unique metabolites in the initiation, maintenance, and progression of each subtype of oral lesion, although the role of the shared metabolites such as nonadecane, disulfide, di-tert-dodecyl, and 2-methylhexacosane in the remodeling of periapical lesions needs further investigation. In our study, the calculated area under the peak value (instead of area percentage) was used, because this is more accurate and, following normalization, allows for better data visualization/representation. In addition, fold changes provide a general overview for the relative analyte abundance, helpful for comparison with future studies.

In the healthy control group, unique metabolites in cluster C include glycolic acid, itaconic acid, and 2-butenedioic acid, all of which are carboxylic acids used to prepare dental pellets (103) for conservative replacement. In cluster D, physiologic signal transduction in healthy pulp tissues is mediated by the neurotransmitter metabolites, like L-aspartic acid and L-glutamic acid (104), in addition to 10-undecynoic acid that maintains the healthy condition due to its antibacterial and antibiofilm activities (105). Unique metabolites in cluster E, like pentadecanoic acid, enhance NK cell activity (106), while 3-dodecanol has antifungal activity (107). Oleic acid in cluster F, which is the most prominent metabolite in healthy pulp tissue, has anti-inflammatory (108), antibacterial (109), and antifungal activities (110) beyond promoting dendritic cell activation (111) to maintain physiologic pulpal conditions. Cluster G palmitic and stearic acids are known to increase NK cell activity (106) and dendritic cell sensitization (111) and induce apoptotic cellular response (112).

In the periapical abscess, cluster H metabolites are mostly known to be correlated to M2 polarization and IL-10 expression; for example, arsenous acid (113) and cholesterol can induce anti-inflammatory M2 polarization (114). These metabolites are present in periapical abscess in lower concentration than in periapical granuloma, indicating that M2 polarization is more inducible in periapical granuloma than in periapical abscess. In cluster I, we found n-butanol, reported to promote NK cell proliferation (115), while butyrate stimulated NK cell cytotoxicity (116) and increased the expression of MMP-9 (117). In the same cluster, alkane metabolites (such as octadecane) stimulate dendritic cells to release IL-6 and IL-10 (118, 119), while isovalerate downregulates the pro-inflammatory polarization of macrophages (120). All metabolites in cluster J are with omega configurations corresponding to fatty acids, dicarboxylic acids, and acetylene. The omega-6 fatty acid, linoelaidic acid, is a pro-inflammatory metabolite (121); dicarboxylic acid calcium oxalate activates human monocytes and enhances the production of IL-8 and IL-10 (122). Finally, the acetylene 17-octadecynoic acid is known to be related to 16-20-HETE, positively correlated with the expression of CYP4F3 (43), VEGF (46), and IL-8 (49).

In the radicular cyst, the alkane and alkene metabolites identified in cluster K are generally known to have a role in inducing cellular membrane flexibility required for optimal cell division and growth (123) and, hence, necessary for radicular cyst expansion. Nonane alkanes and their derivatives in cluster L are considered to be highly hydrophobic lipid molecules (124)and expected to be released during lipophagy. In cluster M, palmitic and stearic acids are significantly correlated with the induction of apoptosis (112), which was identified as the major enrichment pathway in the development of radicular cyst.

In the periapical granuloma, cluster N contains arsenous acid (113), cholesterol (114), octanoic acid (125), L-serine (126), ethanolamine (127), and butanoic acid derivatives (128) that reduce pro-inflammatory M1 or induce anti-inflammatory M2 polarization. Cluster O metabolites included phosphoric acid, which favors chronic inflammation (129), while butylated hydroxytoluene induces M2 macrophage polarization (130). Cluster P predominated in periapical granuloma and clearly differentiated this from other lesions. Despite similar concentrations of ethylene glycol in the periapical abscess and granuloma, the important contributor (L-(+)-lactic acid) was absent in periapical abscess. Interestingly, both L-(+)-lactic acid and ethylene glycol appear to be involved in granuloma formation, a complication associated with skin fillers in plastic surgery (131, 132). This phenomenon is thought to be due to failure in effective phagocytosis leading eventually to granuloma formation (133). Furthermore, ethylene glycol is known to cross-link hyaluronic acid, important for tissue formation/volume (134), while L-lactate polarizes macrophage toward M2 and reduces NK cell cytotoxicity (135–137). Poly-ethylene glycol is also known to favor regenerative M2 macrophage responses (138).

In the healthy pulp tissue, oleic, palmitic, and stearic acids were found to play a crucial role in maintaining a healthy pulpo-dentinal complex (139, 140) with intrinsic antimicrobial activity (141–143). The top enriched metabolic pathways identified in healthy controls are listed in **Table 4**. The highest MCODE in healthy controls reveals that ionotropic receptors N-methyl-D-aspartate (NMDA) (144) and kainate receptors (96) are critical contributors to the maintenance of a healthy pulp (96). Gαq subunit of G protein-coupled receptors (145) appears to activate phospholipase Cβ leading to a significant efflux of Ca^2+^ from the endoplasmic reticulum into the cytosol (146). It has been reported that a high expression of glutamate receptor ionotropic kainate 1 (GRIK1) in healthy pulp tissues correlates with profound tooth sensitivity (147). GRIK2 increases the permeability of Ca^2+^ inside the cell (148), and Ca^2+^ overload results in ischemic cell death (149), which is a phenomenon related to dying tooth pulp during surgical extraction of a healthy tooth. Although *GRIN* genes are encoding NMDA receptor, GRIA encoded the α-amino-3-hydroxy-5-methyl-4-isoxazolepropionic acid (AMPA) receptor, which is another related group of glutamate ionotropic receptor (150). We further identified free fatty acid receptors (FFAR), especially FFAR1 and FFAR3 in healthy pulp tissue, previously reported to bind to palmitic, stearic, and oleic acids (151). These receptors are known to play a key role in maintaining a systemic health condition (140). Activation of group I glutamate metabotropic receptors (GRM), including GRM1 and GRM5, generally induces normal neuronal plasticity (152) (**Figure S2A**).

Although normal healthy pulp was considered to be a sterile space devoid of flora, relatively recent next-generation sequencing data have clearly shown the intriguing presence of bacteria in this tissue space (153) likely to play an important role in maintaining healthy homeostasis. TLR4 on normal odontoblasts (154) binds to LPS, which in turn induces differentiation of human dental pulp stem cells to pulpal odontoblasts (155). In healthy pulp tissue, the predominant immune cells were T-helper cells followed by NK cells, dendritic cells, and CD8 T cells as previously suggested (156–159). Kawashima et al. also reported the presence of NKT cells (160) in healthy pulp tissue, revealing the importance of these in the maintenance of normal pulp homeostasis.

**Figure 8:**
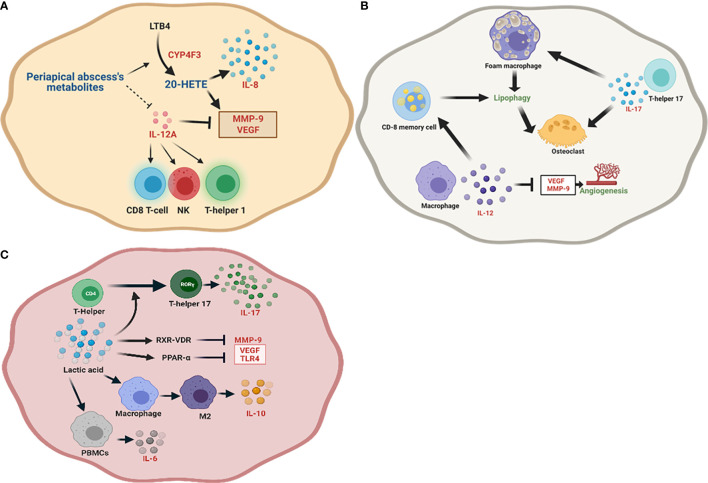
Schematic representation of the identified unique metabolites, enrichment pathways, and proposed genes and their roles in the initiation and progression of each periapical lesion. **(A)** In periapical abscess, the unique metabolic environment stimulates the conversion of leukotriene B4 (LTB4) to 20-HETE and the downregulation of IL-12A. In turn, these upregulate the expression of MMP-9 and VEGF. IL-12A also stimulates the differentiation of naive T cells to T-helper 1 cells and stimulates NK cells and CD8 cytotoxic T lymphocytes. **(B)** In radicular cyst, IL-12 enhances the effector CD8 T cells and memory CD8 T cells. Released IL-12A inhibits VEGF and MMP-9 and hence reduces angiogenesis, while lipophagy is enhanced in foam macrophages. IL-17A released from T-helper 17 directly enhances bone resorption by stimulating osteoclasts. **(C)** In periapical granuloma, L-(+)-lactic acid is the main unique metabolite, which polarizes the conversion of T-helper cells to T-helper 17 through the enhancement of transcription factor RORγ which supports the release of IL-17 and may stimulate RXR-VDR and PPAR-α pathways. RXR-VDR inhibits MMP-9 and PPAR-α negatively regulates VEGF and TLR4. Lactic acid also enhances the polarization of macrophages to the anti-inflammatory M2 phenotype while supporting IL-10 responses.

This study has reported for the first time the role of 20-HETE in the pathogenesis of periapical abscess. It has been found that 17-octadecynoic acid is a partial inhibitor of CYP4Fs (161). In our study, the inhibition of CYP4F3 in abscess was not correlated to 17-octadecynoic acid, the most abundant metabolite significantly identified in the periapical abscess, perhaps due to the significant upregulation of CYP4F3 (**Figure 6A**). This result was consistent with the highest PPI involved in periapical abscess including CYP1A2, CYP2C9, and CYP4F2 (**Figure S2B**). Additionally, the relation between 20-HETE and the significant upregulated genes is highlighted in **Table 4** and **Figure 8A**. Some of the significantly upregulated genes were previously investigated in periapical abscess (7, 162–164), but here, we provide evidence suggesting how they impact metabolic pathways in disease. Our *in silico* immunological profiling of the periapical abscess showed a predominance of T-helper cells followed by dendritic cells. While Ferreira et al. have significantly reported the presence of T-helper cell-related IL-17A production in periapical abscess (163), Harmon et al. demonstrated the presence of mature dendritic in dental abscess compared with healthy dental tissues (165). All the periapical abscess samples in this study were obtained from patients with an Asian ethnicity. Our results reveal that 80% of the recorded mutations in active genes are related to the Asian population, particularly for *MAPT* and *CYP1A2* genes (**Table 2**). In contrast, Salles et al. reported significant polymorphisms in *IL-1B*, *IL-6*, and *IL-8* genes in periapical abscess (166). The metabolic environment of periapical abscess may stimulate the conversion of LTB4 to a less active form, 20-HETE (167), and the downregulation of IL-12A (**Figure 8A**). 20-HETE stimulates the release of IL-8 from endothelial cells by increasing the production of reactive oxygen species and activating the NF-κB pathway (49). Furthermore, both the increase in 20-HETE and the downregulation of IL-12A significantly upregulate the expression of MMP-9 and VEGF (47). This in turn is likely to increase the T-helper cell population, the differentiation of naive T cells into Th1 cells (168), and the stimulation of NK cells (169) and CD8 cytotoxic T lymphocytes (170) (**Figure 8A**).

The abundance of palmitic acid in radicular cyst-derived keratinocytes reveals a high metabolic activity in these cells (171). However, 1-nonadecene, the highest identified unique metabolite in the radicular cyst, has not been reported before and its significance needs to be further studied. While Takata et al. (171) detected the presence of high metabolic activity in radicular cyst, Güler et al. (172) confirmed the significant proliferative capacity in radicular cyst. Excessive cell proliferation in the epithelial lining that may mediate a nutritional deficiency leading to lipophagy (56, 57) is believed to be positively correlated to specific inflammatory mediators such as IL-17 (68) and IL-12A (173) (**Table 4** and **Figures 6F, J****)**. The prominent PPI in radicular cyst was for protein kinase AMP-activated (PRKA) (**Figure S2C**), considered a master activator of autophagy (174). Interestingly, our results reveal the predominance of memory CD8 T cells, followed by pro-inflammatory M1 macrophages and naive CD8 T cells in the radicular cysts. Other studies found that the most abundant immune cells in radicular cysts were memory CD8 T cells (62), in addition to the high polarization toward pro-inflammatory M1 macrophages (175) and a scant presence of CD8 T cells (176). Despite the highest SNPs for POLB were reported in other African population, the Asian population has the highest SNP for PRKAG2, PRKAB2, and F2 (**Table 2**). An evidence of TP63 SNPs in radicular cyst was previously reported by Souza et al. (177). In the radicular cyst, beta-sitosterol and ethanimidic acid were the only unique metabolites found to provide the enriched metabolic pathways as described in **Table 4**. Nevertheless, 1-nonadecene was the highest unique metabolite in the cyst, whose biological and immunological significance has not been reported thus far. In our study, *IL-12A* was the most upregulated gene in radicular cyst compared with other periapical lesions (**Figure 6J**). IL-12 is important in CD8 T-cell clonal expansion in addition to the generation of memory CD8 T cells (65), which in turn stimulate lipophagy (**Figure 8B**). Lipophagy was reported in lipid-laden macrophages (foam macrophages) (59, 60) in odontogenic cyst (63) and in memory CD8 T cells (61). Additionally, IL-12A inhibits VEGF and MMP-9 reducing angiogenesis (66). Furthermore, upregulation of IL-17A may also support lipophagy while enhancing bone resorption *via* osteoclast differentiation (68) (**Figure 8B**).

In periapical granuloma, L-(+)-lactic acid was the highest unique metabolite. Previously, *Lactobacilli* were associated to periapical granuloma (11) and they are exclusively producing L(+)-lactic acid (178). PPI revealed the relation between ESR1, ESRRA, and NR0B1 to periapical granuloma. While granuloma formation is highly dependent on estrogen (179), NR0B1 is related to retinoic acid receptor (180), but data about its relation to periapical granuloma were not reported before. The most predominant immune cells found in periapical granuloma were T-helper cells and M2 macrophages. Previously, T-helper 17 was involved (181) in addition to inflammatory M2 macrophage (175). In our study, datasets of the identified active genes revealed that periapical granulomas have the highest correlation with SNPs for PPARD, TSHR, and SLC16A1 in the African population. In agreement with this, some of the samples included in our study were derived from individuals of Egyptian/North African ethnicity. Lawoyin (182) reported that periapical granuloma accounted for the second most common oral lesion after oral neoplasm in the African population, suggesting that undiscovered SNPs could be associated with the high incidence of periapical granuloma in this population. On the other hand, a significant correlation of SNPs in MMP-1 was reported in periapical granuloma (183). We identified L-(+)-lactic acid as a unique metabolite (**Figure 8C**). This polarizes CD^4+^ T cells toward the T-helper 17 phenotype through the stimulation of the transcription factor RORγ (184). Furthermore, lactic acid stimulates *RXR-VDR* target genes (185) and peroxisome proliferator-activated receptor alpha (PPAR-α) signaling pathway (186). RXR-VDR inhibits MMP-9 (81), while PPAR-α negatively regulates VEGF (187) and TLR4 (88). Additionally, lactic acid polarizes macrophages toward an anti-inflammatory M2 phenotype (188) and favors IL-10 responses (189) and IL-6 release from peripheral blood monocytes (PBMCs) (190) (**Figure 8C**).

This study is the first to investigate the metabolomic and immunological triggers involved in the development and progression of periapical lesions, yet there are few limitations. First, some of the identified metabolites such as 1-noandecene, 5-5-diethylpentadecane, octadecane, 4,4-dimethoxy-2-methyl-2-butanol, and 2,2-dimethyl-1-octanol, that have been searched in PubChem, did not return any related genes (due to no available literature), so these were excluded from further analysis. Second, when using the CIBERSORT tool, the signature matrix or reference list of immune cell genes, LM22 has a limited number of barcode genes. For this reason, we have included another signature genes list from a previous study (39) with a higher number of barcode genes than the LM22, but unfortunately with limited immune cell profiles. In addition, there are no published data about nonadecane, the shared metabolite in our investigated periapical lesions; thus, future investigations are required. Furthermore, future studies are required to validate our gene expression analyses *via* a proteomic approach and investigate the pathogenicity of the identified metabolites on different oral cell lines.

## Conclusion

Periapical lesions of abscess, cyst, and granuloma differ metabolically and immunologically. In comparison with healthy pulp tissue, periapical abscess is predominant with lipid metabolism of 16-20-HETE and 17-octadecynoic acid pathways. Mediators of inflammation and factors involved in their synthesis, like CYP4F3, VEGF, MMP-9, and IL-8, were associated to more acute inflammatory manifestations. Our analysis suggested that radicular cyst correlates with active proliferative responses, characterized by glycogen and lipophagy processes. Immunological markers such as IL-12A may play a critical role in the maintenance of memory CD8 T cells, in radicular cysts. For periapical granuloma, L-(+)-lactic acid and ethylene glycol clustered together and related to granuloma formation. Nuclear receptor transcription factors RXR/VDR and RORγt (which shares sequence similarity with RXR) may be involved in periapical granuloma, supported by substantial expression of IL-17A.

## Data Availability Statement

The datasets presented in this study can be found in online repositories. The names of the repository/repositories and accession number(s) can be found in the article/**Supplementary Material**.

## Ethics Statement

The studies involving human participants were reviewed and approved by Research Ethics Committee at the University of Sharjah (Reference number REC-18-12-17-02-S on 17/02/2019). The patients/participants provided their written informed consent to participate in this study.

## Author Contributions

AA, SS, and RH were responsible for the conception, design, and development of the methodology. AA, SS, and TV shared practical application of the methodology. AA and RH performed the bioinformatics analysis. AA, RH, SS, and LS were responsible for writing and editing the manuscript. RH, SS, and LS supervised the study. SS and RH funded the study. All authors contributed to the article and approved the submitted version.

## Funding

This study was funded by the University of Sharjah (Grant No. 1901110132 to SS and Grant Nos. CoV19-0308, MED001, and 1901090254 to RH).

## Conflict of Interest

The authors declare that the research was conducted in the absence of any commercial or financial relationships that could be construed as a potential conflict of interest.

## Publisher’s Note

All claims expressed in this article are solely those of the authors and do not necessarily represent those of their affiliated organizations, or those of the publisher, the editors and the reviewers. Any product that may be evaluated in this article, or claim that may be made by its manufacturer, is not guaranteed or endorsed by the publisher.
