# Supplementary material for: Comparative Metabolomics Reveals the Microenvironment of Common T-Helper Cells and Differential Immune Cells Linked to Unique Periapical Lesions
Source: Front Immunol. 2021 Sep 3;12:707267. doi: 10.3389/fimmu.2021.707267 (PMC8446658; doi:10.3389/fimmu.2021.707267)
Supplement: Supplementary file 1 [file DataSheet_1.docx]

***Supplementary Materials***

**Supplementary Figures**


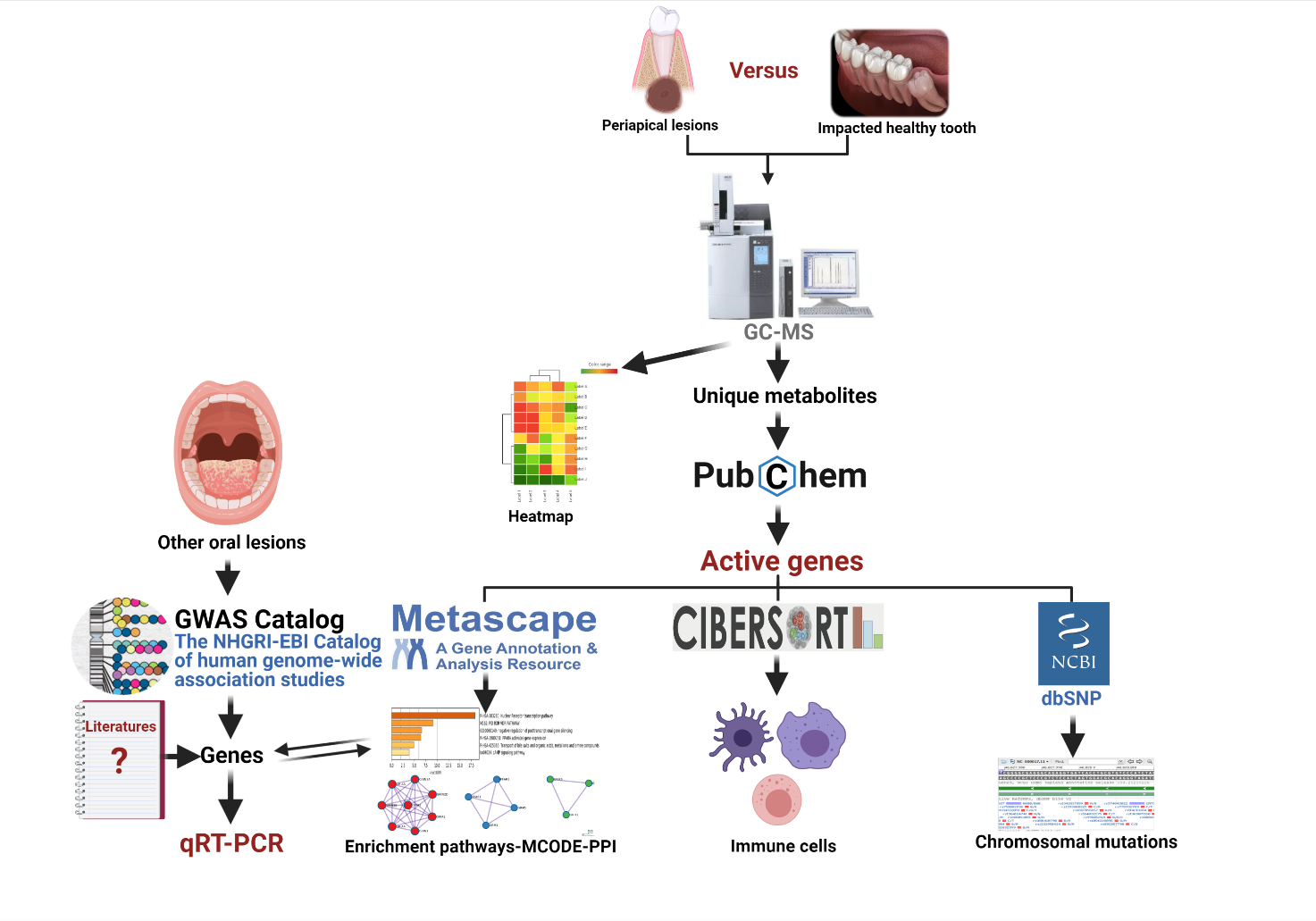


**Supplementary Figure 1.** Flow chart summarized the methodology and procedure employed in this work.


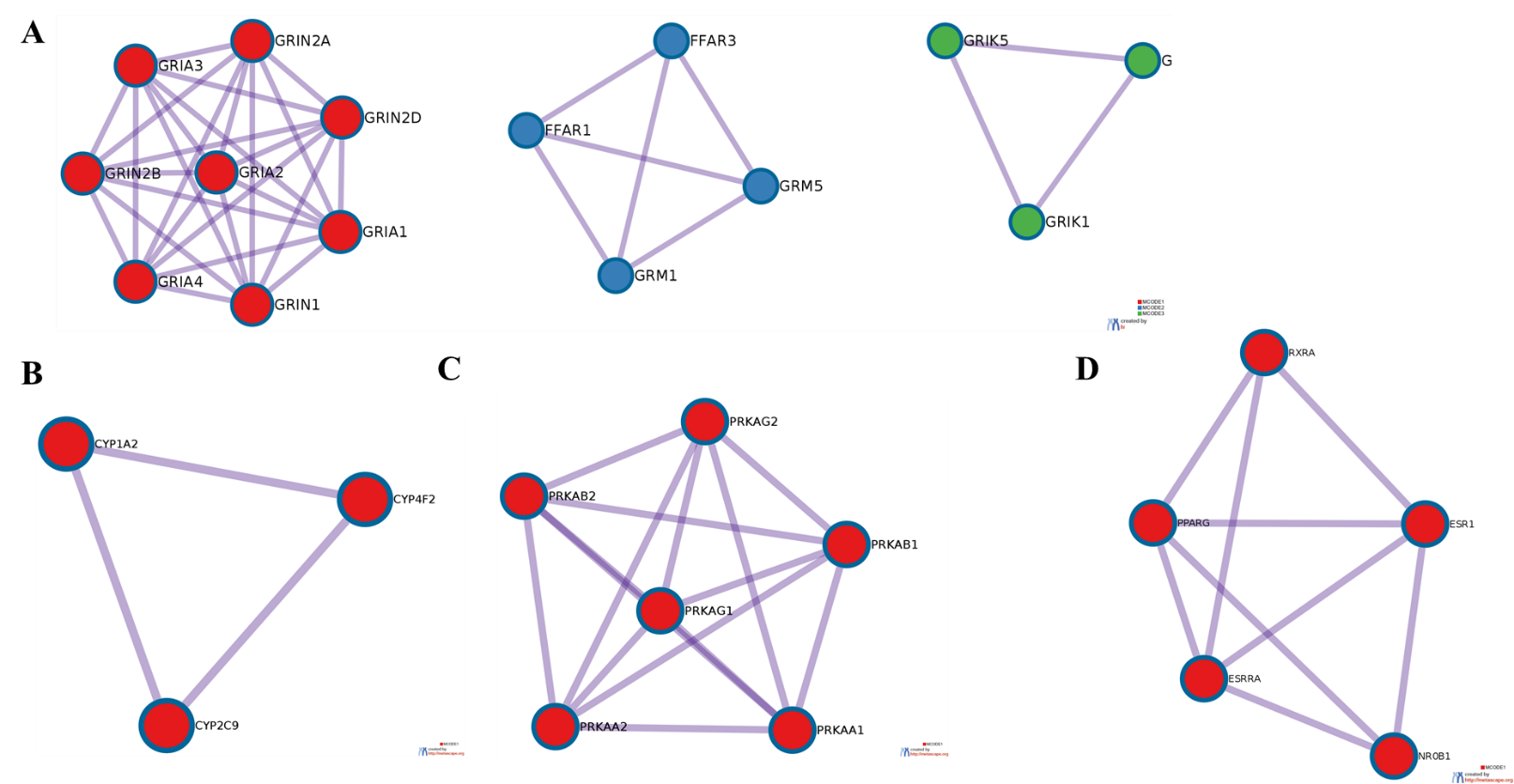


**Supplementary Figure 2.** Protein-protein interaction network identified in the genes using Metascape tool. The highest MCODE 1 represented by red balls, followed by MCODE 2 with blue balls, and then the MCODE 3 with green balls in **(A)** healthy pulp control, **(B)** periapical abscess, **(C**) radicular cyst, and **(D)** periapical granuloma.

**Supplementary Tables**

**Table S1 |** The relation between SNPs of concerned genes and other oral diseases using Genome-wide association (GWAS) approach.

| Disease | Mapped gene | SNP | P-value |
| --- | --- | --- | --- |
| Mouth ulcers | IL-12A | rs76830965 | 4E-483 |
| Mouth ulcers | IL-10, IL-19 | rs1800871 | 6E-236 |
| Mouth ulcers | CCR3 | rs4683205 | 5E-106 |
| Mouth ulcers | NFKBIL1-LTA | rs2516392 | 7E-47 |
| Mouth ulcers | IL-20 | rs2232359 | 3E-45 |
| Mouth ulcers | IL-19 | rs4845140 | 8E-38 |
| Mouth ulcers | IL-19 | rs17015865 | 2E-30 |
| Mouth ulcers | IL-12RB1 | rs2305742 | 5E-28 |
| Mouth ulcers | IL-7R | rs62351974 | 3E-12 |
| Oral cavity cancer | TNF-LTB | rs1800628 | 7E-08 |
| Dental caries | IL-17D | rs735539 | 4E-06 |

**Table S2 |** Primers sequences employed in this study.

| Gene | Accession number | Forward primer (5′~3′) | Reverse primer (5′~3′) | Reference |
| --- | --- | --- | --- | --- |
| 18S rRNA | NR_003286 | TGACTCAACACGGGAAACC | TCGCTCCACCAACTAAGAAC | ([1](#_ENREF_1)) |
| CYP4F3 | NM_000896.3 | CTCATGACCTTGGACAGTC | GTACAGGAGGATCTGCTG | Current study |
| VEGFA | NM_001025366.3 | GCAGAATCATCACGAAGTGGTG ([2](#_ENREF_2)) | CATCAGGGTACTCCTGGAAGAT | Current study |
| MMP-9 | NM_004994.3 | GCCACTACTGTGCCTTTG | CCCTCAGAGAATCGCCAG | Current study |
| IL-8 | NM_000584.4 | GAGAGTGATTGAGAGTGGACCAC | CACAACCCTCTGCACCCAGTTT | ([3](#_ENREF_3)) |
| TLR2 | NM_001318787.2 | AGACCTGTCCCTGAACCCTAT | CGATGGACTTCTAAACCAGCCA | ([4](#_ENREF_4)) |
| IL-17A | NM_002190.3 | CCCCAGTTGATTGGAAGAAA | TTCGTGGGATTGTGATTCCT | ([5](#_ENREF_5)) |
| TLR4 | NM_003266.4 | AGACCTGTCCCTGAACCCTAT | CGATGGACTTCTAAACCAGCCA | ([6](#_ENREF_6)) |
| IL-6 | NM_000600.5 | AGACAGCCACTCACCTCTTCAG | TTCTGCCAGTGCCTCTTTGCTG | ([7](#_ENREF_7)) |
| IL-10 | NM_000572.3 | CCTAACATGCTTCGAGATCTCC | CTCCAGCAAGGACTCCTTTAAC | Current study |
| IL-12A | NM_000882.4 | GCTGGCAGTTATTGATGAGC | GCATGAAGAAGTATGCAGAGC | Current study |

**Table S3 |** Weighted average of area under the curve values of identified metabolites in healthy control and periapical lesions. Each metabolite code reflects its corresponding metabolite in the heatmap.

| Metabolites name | Cluster | Metabolite code |  | PubChem ID | Healthy control | Periapical abscess | Radicular cyst | Periapical granuloma |
| --- | --- | --- | --- | --- | --- | --- | --- | --- |
| 1,3-Propanediol | _ | 1 |  | 10442 | 384896.5 | 0 | 1935167.5 | 280425010 |
| 2-Methyl-1,5-heptadiene-3,4-diol | D | 2 |  | 5366259 | 740536.5 | 0 | 0 | 0 |
| 10-Methylnonadecane | _ | 3 |  | 530070 | 0 | 2032912.5 | 0 | 0 |
| 10-Undecynoic acid | D | 4 |  | 31039 | 1816158.25 | 0 | 0 | 0 |
| 11-Methyldodecanol | L | 5 |  | 33865 | 0 | 0 | 2482140.5 | 0 |
| 17-Octadecynoic acid | J | 6 |  | 1449 | 0 | 10275221.67 | 0 | 0 |
| 2,2-Dimethyl-1-decanol | _ | 7 |  | 520069 | 0 | 1086743 | 0 | 0 |
| 2-Hexyl-1-decanol | BK | 8 |  | 95337 | 17008399.67 | 3330194 | 28330651.33 | 0 |
| 1-Decanol-2-octyl | K | 9 |  | 3084890 | 0 | 0 | 14214052 | 3905239.5 |
| 2-Hexadecyl-1-eicosanol | D | 10 |  | 86602 | 2919175.5 | 0 | 0 | 0 |
| 1-Heneicosanol | HN | 11 |  | 85014 | 0 | 4196386.4 | 0 | 6178691.667 |
| 1-Hexanol, 5-methyl-2-(1-methylethyl) | N | 12 |  | 170582 | 0 | 2979172.75 | 0 | 2946871.5 |
| 1-Monopalmitin | _ | 13 |  | 14900 | 47711891 | 0 | 48848694 | 0 |
| 1-Nonadecene | K | 14 |  | 29075 | 0 | 0 | 22385758.5 | 0 |
| 2,2-Dimethyl-1-octanol | I | 15 |  | 520068 | 0 | 4586227 | 0 | 0 |
| 1-Piperidinecarboxaldehyde | L | 16 |  | 17429 | 0 | 0 | 1826107.5 | 0 |
| 2,6,10-Trimethyltridecane | E | 17 |  | 19774 | 5441245.5 | 0 | 2766870.333 | 0 |
| 2,6-Bis(tert-butyl)phenol | K | 18 |  | 31405 | 5069232 | 0 | 10783128.33 | 0 |
| 2,6-Dimethyl-1-nonen-3-yn-5-ol | _ | 19 |  | 12546097 | 131100173.8 | 0 | 101899385.5 | 0 |
| 2-Bromotetradecane | K | 20 |  | 12798926 | 10111382 | 14397387 | 25892452 | 17460239 |
| 2-Butenedioic acid | C | 21 |  | 444972 | 442878 | 0 | 0 | 0 |
| 2-Hydroxy-3-methylbutyric acid | I | 22 |  | 99823 | 0 | 4044231.333 | 0 | 0 |
| 2-Methylhexacosane | N | 23 |  | 150931 | 0 | 4274416.67 | 8583762.5 | 7019653.5 |
| 2-Methyltetracosane | HN | 24 |  | 527459 | 2607544.5 | 3663993 | 0 | 3670001.667 |
| 2-Propen-1-ol | N | 25 |  | 7858 | 0 | 2257261.67 | 0 | 2370414.333 |
| 2-Propenoic acid | _ | 26 |  | 6581 | 0 | 1199719.667 | 0 | 0 |
| 3-Dodecanol | E | 27 |  | 139108 | 4345450 | 0 | 0 | 0 |
| 3-Ethyl-3-methylheptane | L | 28 |  | 140213 | 0 | 0 | 3274714.5 | 0 |
| 3-Morpholinopropyl isothiocyanate | N | 29 |  | 141745 | 0 | 3276396.6 | 0 | 2532590.5 |
| 3-Pyridinol | C | 30 |  | 7971 | 163885.5 | 0 | 103830 | 0 |
| 4,4-Dimethoxy-2-methyl-2-butanol | I | 31 |  | 4439980 | 0 | 5245000.5 | 0 | 0 |
| 5,5-Diethylheptadecane | BK | 32 |  | 85977275 | 22063896 | 0 | 25289559 | 0 |
| 5,5-Diethylpentadecane | K | 33 |  | 85977274 | 0 | 0 | 15148088 | 0 |
| 6-Ethyl-3-decanol | N | 34 |  | 140584 | 0 | 0 | 0 | 6509451.5 |
| Arsenous acid | HN | 35 |  | 545 | 0 | 3235059 | 0 | 6267341 |
| Behenic acid | N | 36 |  | 8215 | 0 | 1471162.5 | 0 | 3330138 |
| Beta-Sitosterol | _ | 37 |  | 222284 | 0 | 0 | 744493.5 | 0 |
| Butanoic acid, 2-methyl-3-oxo-, ethyl ester | N | 38 |  | 701 | 0 | 2450936 | 0 | 3008429.5 |
| Butylated hydroxytoluene | O | 39 |  | 31404 | 0 | 33222627 | 0 | 31956917.5 |
| Cholesterol | HN | 40 |  | 5997 | 2588099.5 | 3591602.667 | 0 | 5351875 |
| Citric acid | AK | 41 |  | 311 | 14650172.4 | 5871554.25 | 17890573 | 5109831.5 |
| 2,3,5,8-Tetramethyl-decane | _ | 42 |  | 545611 | 0 | 869584.6667 | 0 | 1207382 |
| Decanoic acid | N | 43 |  | 2969 | 0 | 0 | 0 | 17418768.67 |
| Disulfide, di-tert-dodecyl | N | 44 |  | 117981 | 0 | 1612705 | 2629122 | 3866930.5 |
| 1,22-Dibromo-docosane | N | 45 |  | 545960 | 0 | 994077.6667 | 0 | 3559788 |
| 2,6,10-Trimethyl-dodecane | _ | 46 |  | 19773 | 0 | 0 | 0 | 1714545 |
| 2,6,11-Trimethyl-dodecane | HN | 47 |  | 35768 | 2633478.5 | 4036941.333 | 1678813.333 | 5193617.667 |
| Dodecyl nonyl ether | L | 48 |  | 87077689 | 0 | 0 | 2354527 | 0 |
| Eicosane | _ | 49 |  | 8222 | 34342751.25 | 26344896.2 | 72881227.25 | 37603687.53 |
| Ethanimidic acid | L | 50 |  | 178 | 0 | 0 | 3863365 | 0 |
| Ethanolamine | N | 51 |  | 700 | 0 | 1852718 | 0 | 2410220.5 |
| Ether, dodecyl isopropyl | N | 52 |  | 537042 | 0 | 3022339 | 0 | 15103975 |
| Ethylene glycol | P | 53 |  | 174 | 0 | 158096254.5 | 0 | 159420752.5 |
| Glycerol | AK | 54 |  | 753 | 8697106.333 | 3395287.5 | 6437977.333 | 0 |
| Glycerol monostearate | BK | 55 |  | 24699 | 21144201.33 | 0 | 20478652.33 | 0 |
| Glycolic acid | C | 56 |  | 757 | 380732.5 | 0 | 0 | 0 |
| Heneicosane | F | 57 |  | 12403 | 32434742.5 | 0 | 14975509.5 | 0 |
| Heptadecane | K | 58 |  | 12398 | 6926669.5 | 0 | 22304401 | 0 |
| 2,6,10,15-Tetramethyl-heptadecane | N | 59 |  | 41209 | 0 | 1112823 | 0 | 2784300.333 |
| 8-Methyl-heptadecane | L | 60 |  | 292723 | 0 | 0 | 3418340 | 0 |
| Heptadecanoic acid | HN | 61 |  | 10465 | 0 | 4070864.5 | 0 | 6238494.333 |
| Hexadecane | AK | 62 |  | 11006 | 10337094.5 | 3464150.667 | 10250056.33 | 8048929 |
| 2,2,3,3-Tetramethyl-hexane | HN | 63 |  | 26057 | 0 | 3116937 | 0 | 4523731 |
| Isopropyl tetradecyl ether | HN | 64 |  | 54193951 | 0 | 5600590.5 | 0 | 5839169.5 |
| Itaconic acid | C | 65 |  | 811 | 372395.5 | 0 | 0 | 0 |
| L-(+)-Lactic acid | P | 66 |  | 107689 | 0 | 0 | 0 | 254095446.5 |
| L-5-Oxoproline | AK | 67 |  | 7405 | 13033858.75 | 0 | 12282798.33 | 0 |
| Lactic acid | AK | 68 |  | 612 | 12124784.4 | 0 | 11819069.67 | 0 |
| L-Aspartic acid | D | 69 |  | 5960 | 1285060 | 0 | 0 | 0 |
| L-Glutamic acid | D | 70 |  | 33032 | 1178806.5 | 0 | 0 | 0 |
| Lignoceric acid | N | 71 |  | 11197 | 0 | 820249.5 | 0 | 3544605 |
| Linoelaidic acid | J | 72 |  | 5282457 | 0 | 12849289 | 0 | 7894288 |
| L-Serine | N | 73 |  | 5951 | 0 | 4270304.25 | 0 | 2760831 |
| Mandelic acid | _ | 74 |  | 1292 | 44567213 | 0 | 115581166.3 | 0 |
| Methoxyacetic acid, 4-hexadecyl ester | L | 75 |  | 545959 | 0 | 0 | 6215609.667 | 0 |
| Methyl isovalerate | I | 76 |  | 11160 | 0 | 2813623.5 | 0 | 0 |
| Myristic acid | _ | 77 |  | 11005 | 8107089 | 4207986.6 | 15103600.5 | 5972904.25 |
| Nonadecane | N | 78 |  | 12401 | 0 | 8580936.75 | 6391342.6 | 13731220 |
| Nonadecyl pentafluoropropionate | L | 79 |  | 91693316 | 0 | 0 | 3235751.333 | 0 |
| 5-Butyl-nonane | L | 80 |  | 300476 | 0 | 0 | 4298562.5 | 0 |
| 5-Methyl-5-propyl-nonane | F | 81 |  | 551397 | 42030758 | 0 | 19309954 | 0 |
| Nonanoic acid | _ | 82 |  | 8158 | 0 | 1211198.667 | 0 | 1620775 |
| Nonyl tetracosyl ether | L | 83 |  | 87077463 | 0 | 0 | 4118983.5 | 0 |
| Octacosane | I | 84 |  | 12408 | 0 | 3732742.667 | 0 | 0 |
| Octadecane | K | 85 |  | 11635 | 0 | 0 | 10974712.5 | 0 |
| 2-Methyl-octadecane | N | 86 |  | 129689219 | 0 | 2649439.5 | 0 | 10032514.5 |
| 3-Ethyl-5-(2-ethylbutyl)-octadecane | I | 87 |  | 292285 | 0 | 2834042.5 | 0 | 0 |
| Octanoic acid | N | 88 |  | 379 | 0 | 0 | 0 | 10311152 |
| Oleic acid | F | 89 |  | 445639 | 71066109.67 | 0 | 0 | 0 |
| Oxalic acid | J | 90 |  | 971 | 0 | 17352324.33 | 0 | 3082977 |
| Palmitic acid | GM | 91 |  | 985 | 410415474.8 | 103162193.4 | 380895169 | 88535339.25 |
| Pentadecanoic acid | E | 92 |  | 13849 | 5510837.333 | 0 | 0 | 0 |
| Petroselinic acid | N | 93 |  | 5281125 | 0 | 0 | 0 | 12322983.5 |
| Phosphoric acid | O | 94 |  | 1004 | 0 | 19291206 | 0 | 51440521.5 |
| Stearic acid | GM | 95 |  | 5281 | 356526268.8 | 103414678.4 | 308783682.8 | 140731568.8 |
| Sulfurous acid, 2-propyl tetradecyl ester | K | 96 |  | 6420356 | 0 | 3745294.5 | 16613336.5 | 0 |
| Sulfurous acid, 2-propyl tridecyl ester | N | 97 |  | 6420355 | 0 | 0 | 0 | 4060245 |
| Sulfurous acid, octadecyl 2-propyl ester | F | 98 |  | 6420358 | 24836630.5 | 0 | 0 | 4736048 |
| 1-Iodo-tetracosane | _ | 99 |  | 11282694 | 0 | 2308911.5 | 0 | 0 |
| 2,6,10-Trimethyl-tetradecane | _ | 100 |  | 85785 | 0 | 0 | 0 | 1438330.5 |
| Tetrapentacontane | _ | 101 |  | 521846 | 18876100.33 | 0 | 59359847.33 | 0 |
| 1,54-Dibromotetrapentacontane | E | 102 |  | 545963 | 8676554.5 | 0 | 0 | 0 |
| Urea | N | 103 |  | 1176 | 747690.6667 | 3085685.8 | 2253928.667 | 7808819.5 |

**Table S4 |** Area under the curve of each metabolite in healthy control and periapical lesions samples. Metabolites are represented as codes in the respective heatmap.

| Metabolites name | Cluster | Metabolites code | PubChem ID | Healthy control 1 | Healthy control 2 | Healthy control 3 | Healthy control 4 | Healthy control 5 | Periapical abscess 1 | Periapical abscess 2 | Periapical abscess 3 | Periapical abscess 4 | Periapical abscess 5 | Radicular cyst 1 | Radicular cyst 2 | Radicular cyst 3 | Radicular cyst 4 | Periapical granuloma 1 | Periapical granuloma 2 | Periapical granuloma 3 | Periapical granuloma 4 |
| --- | --- | --- | --- | --- | --- | --- | --- | --- | --- | --- | --- | --- | --- | --- | --- | --- | --- | --- | --- | --- | --- |
| 1,3-Propanediol | _ | 1 | 10442 | 0 | 307788 | 0 | 462005 | 0 | 0 | 0 | 0 | 0 | 0 | 0 | 1238734 | 2631601 | 0 | 2.21E+08 | 0 | 0 | 3.39E+08 |
| 2-Methyl-1,5-heptadiene-3,4-diol | D | 2 | 5366259 | 0 | 0 | 683325 | 0 | 797748 | 0 | 0 | 0 | 0 | 0 | 0 | 0 | 0 | 0 | 0 | 0 | 0 | 0 |
| 10-Methylnonadecane | _ | 3 | 530070 | 0 | 0 | 0 | 0 | 0 | 2353423 | 0 | 0 | 0 | 1712402 | 0 | 0 | 0 | 0 | 0 | 0 | 0 | 0 |
| 10-Undecynoic acid | D | 4 | 31039 | 562421 | 3917283 | 1769083 | 1015846 | 0 | 0 | 0 | 0 | 0 | 0 | 0 | 0 | 0 | 0 | 0 | 0 | 0 | 0 |
| 11-Methyldodecanol | L | 5 | 33865 | 0 | 0 | 0 | 0 | 0 | 0 | 0 | 0 | 0 | 0 | 661264 | 4303017 | 0 | 0 | 0 | 0 | 0 | 0 |
| 17-Octadecynoic acid | J | 6 | 1449 | 0 | 0 | 0 | 0 | 0 | 0 | 11450185 | 0 | 13692833 | 5682647 | 0 | 0 | 0 | 0 | 0 | 0 | 0 | 0 |
| 2,2-Dimethyl-1-decanol | _ | 7 | 520069 | 0 | 0 | 0 | 0 | 0 | 0 | 0 | 291938 | 1881548 | 0 | 0 | 0 | 0 | 0 | 0 | 0 | 0 | 0 |
| 2-Hexyl-1-decanol | BK | 8 | 95337 | 7362131 | 0 | 22468125 | 21194943 | 0 | 3907205 | 0 | 0 | 2753183 | 0 | 57061805 | 21779081 | 0 | 6151068 | 0 | 0 | 0 | 0 |
| 1-Decanol-2-octyl | K | 9 | 3084890 | 0 | 0 | 0 | 0 | 0 | 0 | 0 | 0 | 0 | 0 | 26843995 | 0 | 0 | 1584109 | 0 | 4934888 | 2875591 | 0 |
| 2-Hexadecyl-1-eicosanol | D | 10 | 86602 | 0 | 0 | 586550 | 5251801 | 0 | 0 | 0 | 0 | 0 | 0 | 0 | 0 | 0 | 0 | 0 | 0 | 0 | 0 |
| 1-Heneicosanol | HN | 11 | 85014 | 0 | 0 | 0 | 0 | 0 | 5389849 | 1271191 | 5455459 | 6515987 | 2349446 | 0 | 0 | 0 | 0 | 0 | 8269178 | 5505783 | 4761114 |
| 1-Hexanol, 5-methyl-2-(1-methylethyl) | N | 12 | 170582 | 0 | 0 | 0 | 0 | 0 | 1550109 | 0 | 3867178 | 543955 | 5955449 | 0 | 0 | 0 | 0 | 0 | 954281 | 4939462 | 0 |
| 1-Monopalmitin | _ | 13 | 14900 | 2490505 | 39587696 | 55658929 | 0 | 93110434 | 0 | 0 | 0 | 0 | 0 | 80433626 | 59353459 | 6758997 | 0 | 0 | 0 | 0 | 0 |
| 1-Nonadecene | K | 14 | 29075 | 0 | 0 | 0 | 0 | 0 | 0 | 0 | 0 | 0 | 0 | 36839065 | 7932452 | 0 | 0 | 0 | 0 | 0 | 0 |
| 2,2-Dimethyl-1-octanol | I | 15 | 520068 | 0 | 0 | 0 | 0 | 0 | 4017000 | 0 | 5155454 | 0 | 0 | 0 | 0 | 0 | 0 | 0 | 0 | 0 | 0 |
| 1-Piperidinecarboxaldehyde | L | 16 | 17429 | 0 | 0 | 0 | 0 | 0 | 0 | 0 | 0 | 0 | 0 | 2256223 | 1395992 | 0 | 0 | 0 | 0 | 0 | 0 |
| 2,6,10-Trimethyltridecane | E | 17 | 19774 | 0 | 3502171 | 0 | 7380320 | 0 | 0 | 0 | 0 | 0 | 0 | 0 | 3684106 | 3081303 | 1535202 | 0 | 0 | 0 | 0 |
| 2,6-Bis(tert-butyl)phenol | K | 18 | 31405 | 0 | 6411225 | 3463761 | 5813926 | 4588016 | 0 | 0 | 0 | 0 | 0 | 14320099 | 5782356 | 12246930 | 0 | 0 | 0 | 0 | 0 |
| 2,6-Dimethyl-1-nonen-3-yn-5-ol | _ | 19 | 12546097 | 0 | 1.15E+08 | 94404947 | 1.19E+08 | 1.96E+08 | 0 | 0 | 0 | 0 | 0 | 1.5E+08 | 53873609 | 0 | 0 | 0 | 0 | 0 | 0 |
| 2-Bromotetradecane | K | 20 | 12798926 | 12577951 | 0 | 7644813 | 0 | 0 | 20524795 | 10081041 | 15693544 | 8186072 | 17501483 | 0 | 0 | 0 | 25892452 | 10935983 | 23285327 | 18159407 | 0 |
| 2-Butenedioic acid | C | 21 | 444972 | 0 | 744619 | 141137 | 0 | 0 | 0 | 0 | 0 | 0 | 0 | 0 | 0 | 0 | 0 | 0 | 0 | 0 | 0 |
| 2-Hydroxy-3-methylbutyric acid | I | 22 | 99823 | 0 | 0 | 0 | 0 | 0 | 0 | 2148294 | 6761144 | 3223256 | 0 | 0 | 0 | 0 | 0 | 0 | 0 | 0 | 0 |
| 2-Methylhexacosane | N | 23 | 150931 | 0 | 0 | 0 | 0 | 0 | 8307876 | 2052602 | 0 | 2462772 | 0 | 5833148 | 0 | 0 | 11334377 | 0 | 4655521 | 9383786 | 0 |
| 2-Methyltetracosane | HN | 24 | 527459 | 2158131 | 0 | 3056958 | 0 | 0 | 5514092 | 0 | 1813894 | 0 | 0 | 0 | 0 | 0 | 0 | 2580201 | 2781442 | 5648362 | 0 |
| 2-Propen-1-ol | N | 25 | 7858 | 0 | 0 | 0 | 0 | 0 | 2678574 | 2120118 | 0 | 1973093 | 0 | 0 | 0 | 0 | 0 | 2197851 | 2631002 | 2282390 | 0 |
| 2-Propenoic acid | _ | 26 | 6581 | 0 | 0 | 0 | 0 | 0 | 1492487 | 1048767 | 0 | 1057905 | 0 | 0 | 0 | 0 | 0 | 0 | 0 | 0 | 0 |
| 3-Dodecanol | E | 27 | 139108 | 4352941 | 0 | 0 | 4337959 | 0 | 0 | 0 | 0 | 0 | 0 | 0 | 0 | 0 | 0 | 0 | 0 | 0 | 0 |
| 3-Ethyl-3-methylheptane | L | 28 | 140213 | 0 | 0 | 0 | 0 | 0 | 0 | 0 | 0 | 0 | 0 | 0 | 0 | 1700831 | 4848598 | 0 | 0 | 0 | 0 |
| 3-Morpholinopropyl isothiocyanate | N | 29 | 141745 | 0 | 0 | 0 | 0 | 0 | 2622808 | 2476868 | 5128164 | 2555423 | 3598720 | 0 | 0 | 0 | 0 | 1695246 | 2399543 | 2453940 | 3581633 |
| 3-Pyridinol | C | 30 | 7971 | 0 | 0 | 137658 | 190113 | 0 | 0 | 0 | 0 | 0 | 0 | 0 | 103830 | 0 | 0 | 0 | 0 | 0 | 0 |
| 4,4-Dimethoxy-2-methyl-2-butanol | I | 31 | 4439980 | 0 | 0 | 0 | 0 | 0 | 4789833 | 0 | 0 | 0 | 5700168 | 0 | 0 | 0 | 0 | 0 | 0 | 0 | 0 |
| 5,5-Diethylheptadecane | BK | 32 | 85977275 | 0 | 0 | 0 | 6734543 | 37393249 | 0 | 0 | 0 | 0 | 0 | 40918682 | 9660436 | 0 | 0 | 0 | 0 | 0 | 0 |
| 5,5-Diethylpentadecane | K | 33 | 85977274 | 0 | 0 | 0 | 0 | 0 | 0 | 0 | 0 | 0 | 0 | 26641397 | 3654779 | 0 | 0 | 0 | 0 | 0 | 0 |
| 6-Ethyl-3-decanol | N | 34 | 140584 | 0 | 0 | 0 | 0 | 0 | 0 | 0 | 0 | 0 | 0 | 0 | 0 | 0 | 0 | 0 | 0 | 4444249 | 8574654 |
| Arsenous acid | HN | 35 | 545 | 0 | 0 | 0 | 0 | 0 | 2614875 | 3855243 | 0 | 0 | 0 | 0 | 0 | 0 | 0 | 0 | 4324714 | 2707363 | 11769946 |
| Behenic acid | N | 36 | 8215 | 0 | 0 | 0 | 0 | 0 | 2059103 | 0 | 0 | 0 | 883222 | 0 | 0 | 0 | 0 | 5329821 | 3557136 | 2649478 | 1784117 |
| Beta-Sitosterol | _ | 37 | 222284 | 0 | 0 | 0 | 0 | 0 | 0 | 0 | 0 | 0 | 0 | 906233 | 582754 | 0 | 0 | 0 | 0 | 0 | 0 |
| Butanoic acid, 2-methyl-3-oxo-, ethyl ester | N | 38 | 701 | 0 | 0 | 0 | 0 | 0 | 0 | 2392142 | 2660019 | 0 | 2300647 | 0 | 0 | 0 | 0 | 2866188 | 3150671 | 0 | 0 |
| Butylated Hydroxytoluene | O | 39 | 31404 | 0 | 0 | 0 | 0 | 0 | 44179050 | 14301771 | 46244123 | 26217374 | 35170817 | 0 | 0 | 0 | 0 | 33810377 | 16176252 | 31529558 | 46311483 |
| Cholesterol | HN | 40 | 5997 | 0 | 0 | 1187806 | 0 | 3988393 | 2861071 | 3911769 | 0 | 4001968 | 0 | 0 | 0 | 0 | 0 | 0 | 5189173 | 5514577 | 0 |
| Citric acid | AK | 41 | 311 | 5947190 | 10913248 | 10211564 | 12755248 | 33423612 | 7839189 | 3131537 | 6468345 | 6047146 | 0 | 27896672 | 7884474 | 0 | 0 | 0 | 4320986 | 5898677 | 0 |
| 2,3,5,8-Tetramethyl-decane | _ | 42 | 545611 | 0 | 0 | 0 | 0 | 0 | 757644 | 620563 | 0 | 1230547 | 0 | 0 | 0 | 0 | 0 | 1207382 | 0 | 0 | 0 |
| Decanoic acid | N | 43 | 2969 | 0 | 0 | 0 | 0 | 0 | 0 | 0 | 0 | 0 | 0 | 0 | 0 | 0 | 0 | 11941944 | 24514466 | 15799896 | 0 |
| Disulfide, di-tert-dodecyl | N | 44 | 117981 | 0 | 0 | 0 | 0 | 0 | 1199502 | 1852997 | 0 | 1785616 | 0 | 875708 | 0 | 0 | 4382536 | 0 | 2221597 | 5512264 | 0 |
| 1,22-Dibromo-docosane | N | 45 | 545960 | 0 | 0 | 0 | 0 | 0 | 0 | 1209018 | 1506493 | 0 | 266722 | 0 | 0 | 0 | 0 | 3603619 | 0 | 0 | 3515957 |
| 2,6,10-Trimethyl-dodecane | _ | 46 | 19773 | 0 | 0 | 0 | 0 | 0 | 0 | 0 | 0 | 0 | 0 | 0 | 0 | 0 | 0 | 0 | 843107 | 0 | 2585983 |
| 2,6,11-Trimethyl-dodecane | HN | 47 | 35768 | 4731855 | 0 | 0 | 535102 | 0 | 4734348 | 2392633 | 0 | 0 | 4983843 | 586232 | 1051529 | 0 | 3398679 | 0 | 4217286 | 6882083 | 4481484 |
| Dodecyl nonyl ether | L | 48 | 87077689 | 0 | 0 | 0 | 0 | 0 | 0 | 0 | 0 | 0 | 0 | 0 | 994990 | 0 | 3714064 | 0 | 0 | 0 | 0 |
| Eicosane | _ | 49 | 8222 | 0 | 49368798 | 987956 | 17189297 | 69824954 | 60939718 | 8794125 | 38100244 | 14313524 | 9576870 | 1.53E+08 | 28616531 | 40908341 | 69184485 | 11971675 | 57684041 | 5548936 | 75210098 |
| Ethanimidic acid | L | 50 | 178 | 0 | 0 | 0 | 0 | 0 | 0 | 0 | 0 | 0 | 0 | 7723239 | 0 | 3491 | 0 | 0 | 0 | 0 | 0 |
| Ethanolamine | N | 51 | 700 | 0 | 0 | 0 | 0 | 0 | 2669486 | 1035950 | 0 | 0 | 0 | 0 | 0 | 0 | 0 | 1625536 | 3653562 | 2361124 | 2000660 |
| Ether, dodecyl isopropyl | N | 52 | 537042 | 0 | 0 | 0 | 0 | 0 | 3920761 | 0 | 3230499 | 1915757 | 0 | 0 | 0 | 0 | 0 | 0 | 0 | 15981266 | 14226684 |
| Ethylene glycol | P | 53 | 174 | 0 | 0 | 0 | 0 | 0 | 1.89E+08 | 1.45E+08 | 0 | 1.5E+08 | 1.48E+08 | 0 | 0 | 0 | 0 | 168576 | 1.88E+08 | 1.75E+08 | 2.74E+08 |
| Glycerol | AK | 54 | 753 | 0 | 0 | 7345985 | 10097190 | 8648144 | 3544744 | 0 | 0 | 3245831 | 0 | 7423028 | 7389912 | 4500992 | 0 | 0 | 0 | 0 | 0 |
| Glycerol monostearate | BK | 55 | 24699 | 0 | 9534264 | 25507211 | 0 | 28391129 | 0 | 0 | 0 | 0 | 0 | 25761474 | 17567864 | 18106619 | 0 | 0 | 0 | 0 | 0 |
| Glycolic acid | C | 56 | 757 | 0 | 0 | 0 | 331177 | 430288 | 0 | 0 | 0 | 0 | 0 | 0 | 0 | 0 | 0 | 0 | 0 | 0 | 0 |
| Heneicosane | F | 57 | 12403 | 0 | 31603181 | 11336270 | 11825298 | 74974221 | 0 | 0 | 0 | 0 | 0 | 41950480 | 2974115 | 1933.5 | 0 | 0 | 0 | 0 | 0 |
| Heptadecane | K | 58 | 12398 | 0 | 0 | 2458022 | 11395317 | 0 | 0 | 0 | 0 | 0 | 0 | 36564510 | 8044292 | 0 | 0 | 0 | 0 | 0 | 0 |
| 2,6,10,15-Tetramethyl-heptadecane | N | 59 | 41209 | 0 | 0 | 0 | 0 | 0 | 0 | 743930 | 0 | 1481716 | 0 | 0 | 0 | 0 | 0 | 3376783 | 2117665 | 2858453 | 0 |
| 8-Methyl-heptadecane | L | 60 | 292723 | 0 | 0 | 0 | 0 | 0 | 0 | 0 | 0 | 0 | 0 | 4789957 | 2046723 | 0 | 0 | 0 | 0 | 0 | 0 |
| Heptadecanoic acid | HN | 61 | 10465 | 0 | 0 | 0 | 0 | 0 | 5868490 | 2273239 | 0 | 0 | 0 | 0 | 0 | 0 | 0 | 5343224 | 7945356 | 5426903 | 0 |
| Hexadecane | AK | 62 | 11006 | 11838955 | 8835234 | 0 | 0 | 0 | 4347162 | 1617183 | 0 | 4428107 | 0 | 6033651 | 8157906 | 0 | 16558612 | 5822437 | 7433210 | 10891140 | 0 |
| 2,2,3,3-Tetramethyl-hexane | HN | 63 | 26057 | 0 | 0 | 0 | 0 | 0 | 0 | 5976337 | 0 | 0 | 257537 | 0 | 0 | 0 | 0 | 0 | 4523731 | 0 | 0 |
| Isopropyl tetradecyl ether | HN | 64 | 54193951 | 0 | 0 | 0 | 0 | 0 | 17670196 | 2140050 | 1309090 | 1283026 | 0 | 0 | 0 | 0 | 0 | 0 | 4048187 | 7630152 | 0 |
| Itaconic acid | C | 65 | 811 | 0 | 525957 | 218834 | 0 | 0 | 0 | 0 | 0 | 0 | 0 | 0 | 0 | 0 | 0 | 0 | 0 | 0 | 0 |
| L-(+)-Lactic acid | P | 66 | 107689 | 0 | 0 | 0 | 0 | 0 | 0 | 0 | 0 | 0 | 0 | 0 | 0 | 0 | 0 | 0 | 2.41E+08 | 2.67E+08 | 0 |
| L-5-Oxoproline | AK | 67 | 7405 | 0 | 24671739 | 12972818 | 8400605 | 6090273 | 0 | 0 | 0 | 0 | 0 | 17472935 | 9920746 | 9454714 | 0 | 0 | 0 | 0 | 0 |
| Lactic Acid | AK | 68 | 612 | 2605416 | 11270949 | 15232244 | 11975565 | 19539748 | 0 | 0 | 0 | 0 | 0 | 20196962 | 10664780 | 4595467 | 0 | 0 | 0 | 0 | 0 |
| L-Aspartic acid | D | 69 | 5960 | 0 | 1846111 | 724009 | 0 | 0 | 0 | 0 | 0 | 0 | 0 | 0 | 0 | 0 | 0 | 0 | 0 | 0 | 0 |
| L-Glutamic acid | D | 70 | 33032 | 0 | 0 | 842416 | 1515197 | 0 | 0 | 0 | 0 | 0 | 0 | 0 | 0 | 0 | 0 | 0 | 0 | 0 | 0 |
| Lignoceric acid | N | 71 | 11197 | 0 | 0 | 0 | 0 | 0 | 691947 | 0 | 948552 | 0 | 0 | 0 | 0 | 0 | 0 | 7508565 | 1833089 | 0 | 1292161 |
| Linoelaidic acid | J | 72 | 5282457 | 0 | 0 | 0 | 0 | 0 | 13281972 | 0 | 12416606 | 0 | 0 | 0 | 0 | 0 | 0 | 1677209 | 0 | 14111367 | 0 |
| L-Serine | N | 73 | 5951 | 0 | 0 | 0 | 0 | 0 | 2297135 | 2566100 | 0 | 1435172 | 10782810 | 0 | 0 | 0 | 0 | 0 | 2830589 | 2691073 | 0 |
| Mandelic acid | _ | 74 | 1292 | 1181378 | 99318725 | 14472878 | 57528564 | 50334520 | 0 | 0 | 0 | 0 | 0 | 22544965 | 88772868 | 2.35E+08 | 0 | 0 | 0 | 0 | 0 |
| Methoxyacetic acid, 4-hexadecyl ester | L | 75 | 545959 | 0 | 0 | 0 | 0 | 0 | 0 | 0 | 0 | 0 | 0 | 0 | 273356 | 17086875 | 1286598 | 0 | 0 | 0 | 0 |
| Methyl isovalerate | I | 76 | 11160 | 0 | 0 | 0 | 0 | 0 | 3212085 | 0 | 0 | 2415162 | 0 | 0 | 0 | 0 | 0 | 0 | 0 | 0 | 0 |
| Myristic acid | _ | 77 | 11005 | 5382641 | 0 | 10202756 | 8735870 | 0 | 4819624 | 2520326 | 5321816 | 4497882 | 3880285 | 23039173 | 7168028 | 0 | 0 | 5208102 | 5629692 | 5234504 | 7819319 |
| Nonadecane | N | 78 | 12401 | 0 | 0 | 0 | 0 | 0 | 9058110 | 0 | 10174215 | 4922097 | 10169325 | 0 | 6391343 | 0 | 0 | 4670621 | 36961312 | 6572657 | 6720290 |
| Nonadecyl pentafluoropropionate | L | 79 | 91693316 | 0 | 0 | 0 | 0 | 0 | 0 | 0 | 0 | 0 | 0 | 1778674 | 7476687 | 451893 | 0 | 0 | 0 | 0 | 0 |
| 5-Butyl-nonane | L | 80 | 300476 | 0 | 0 | 0 | 0 | 0 | 0 | 0 | 0 | 0 | 0 | 7706128 | 890997 | 0 | 0 | 0 | 0 | 0 | 0 |
| 5-Methyl-5-propyl-nonane | F | 81 | 551397 | 0 | 0 | 9173284 | 0 | 74888232 | 0 | 0 | 0 | 0 | 0 | 34516006 | 4103902 | 0 | 0 | 0 | 0 | 0 | 0 |
| Nonanoic acid | _ | 82 | 8158 | 0 | 0 | 0 | 0 | 0 | 855602 | 558858 | 0 | 2219136 | 0 | 0 | 0 | 0 | 0 | 2444042 | 797508 | 0 | 0 |
| Nonyl tetracosyl ether | L | 83 | 87077463 | 0 | 0 | 0 | 0 | 0 | 0 | 0 | 0 | 0 | 0 | 618278 | 7619689 | 0 | 0 | 0 | 0 | 0 | 0 |
| Octacosane | I | 84 | 12408 | 0 | 0 | 0 | 0 | 0 | 2913067 | 0 | 0 | 3428235 | 4856926 | 0 | 0 | 0 | 0 | 0 | 0 | 0 | 0 |
| Octadecane | K | 85 | 11635 | 0 | 0 | 0 | 0 | 0 | 0 | 0 | 0 | 0 | 0 | 18990434 | 2958991 | 0 | 0 | 0 | 0 | 0 | 0 |
| 2-Methyl-octadecane | N | 86 | 129689219 | 0 | 0 | 0 | 0 | 0 | 1531006 | 0 | 0 | 0 | 3767873 | 0 | 0 | 0 | 0 | 0 | 13875585 | 6189444 | 0 |
| 3-Ethyl-5-(2-ethylbutyl)-octadecane | I | 87 | 292285 | 0 | 0 | 0 | 0 | 0 | 1687273 | 3980812 | 0 | 0 | 0 | 0 | 0 | 0 | 0 | 0 | 0 | 0 | 0 |
| Octanoic acid | N | 88 | 379 | 0 | 0 | 0 | 0 | 0 | 0 | 0 | 0 | 0 | 0 | 0 | 0 | 0 | 0 | 7674588 | 0 | 12947716 | 0 |
| Oleic Acid | F | 89 | 445639 | 0 | 33682620 | 42106416 | 0 | 1.37E+08 | 0 | 0 | 0 | 0 | 0 | 0 | 0 | 0 | 0 | 0 | 0 | 0 | 0 |
| Oxalic acid | J | 90 | 971 | 0 | 0 | 0 | 0 | 0 | 0 | 12198006 | 0 | 15939418 | 23919549 | 0 | 0 | 0 | 0 | 2987536 | 3178418 | 0 | 0 |
| Palmitic Acid | GM | 91 | 985 | 88239060 | 4.36E+08 | 4.86E+08 | 4.04E+08 | 6.38E+08 | 1.16E+08 | 65916642 | 1.09E+08 | 1.11E+08 | 1.13E+08 | 6.3E+08 | 3.39E+08 | 4E+08 | 1.54E+08 | 1.2E+08 | 1.16E+08 | 1.06E+08 | 12266514 |
| Pentadecanoic acid | E | 92 | 13849 | 0 | 7129733 | 4874394 | 4528385 | 0 | 0 | 0 | 0 | 0 | 0 | 0 | 0 | 0 | 0 | 0 | 0 | 0 | 0 |
| Petroselinic acid | N | 93 | 5281125 | 0 | 0 | 0 | 0 | 0 | 0 | 0 | 0 | 0 | 0 | 0 | 0 | 0 | 0 | 8926986 | 15718981 | 0 | 0 |
| Phosphoric acid | O | 94 | 1004 | 0 | 0 | 0 | 0 | 0 | 23880527 | 12562552 | 25817359 | 15461596 | 18733996 | 0 | 0 | 0 | 0 | 22903262 | 32124418 | 28069266 | 1.23E+08 |
| Stearic acid | GM | 95 | 5281 | 73668242 | 3.52E+08 | 4.53E+08 | 3.61E+08 | 5.42E+08 | 1.12E+08 | 73441954 | 1.19E+08 | 1.09E+08 | 1.04E+08 | 5.06E+08 | 3.02E+08 | 2.71E+08 | 1.56E+08 | 2.1E+08 | 1.17E+08 | 95794144 | 1.41E+08 |
| Sulfurous acid, 2-propyl tetradecyl ester | K | 96 | 6420356 | 0 | 0 | 0 | 0 | 0 | 0 | 3040918 | 4449671 | 0 | 0 | 0 | 0 | 30427994 | 2798679 | 0 | 0 | 0 | 0 |
| Sulfurous acid, 2-propyl tridecyl ester | N | 97 | 6420355 | 0 | 0 | 0 | 0 | 0 | 0 | 0 | 0 | 0 | 0 | 0 | 0 | 0 | 0 | 4853050 | 2336215 | 25623 | 9026092 |
| Sulfurous acid, octadecyl 2-propyl ester | F | 98 | 6420358 | 3840967 | 0 | 0 | 0 | 45832294 | 0 | 0 | 0 | 0 | 0 | 0 | 0 | 0 | 0 | 0 | 5278550 | 4193546 | 0 |
| 1-Iodo-tetracosane | _ | 99 | 11282694 | 0 | 0 | 0 | 0 | 0 | 0 | 0 | 1519157 | 3098666 | 0 | 0 | 0 | 0 | 0 | 0 | 0 | 0 | 0 |
| 2,6,10-Trimethyl-tetradecane | _ | 100 | 85785 | 0 | 0 | 0 | 0 | 0 | 0 | 0 | 0 | 0 | 0 | 0 | 0 | 0 | 0 | 222984 | 2653677 | 0 | 0 |
| Tetrapentacontane | _ | 101 | 521846 | 0 | 0 | 5201174 | 2942326 | 48484801 | 0 | 0 | 0 | 0 | 0 | 1.16E+08 | 7495607 | 54625233 | 0 | 0 | 0 | 0 | 0 |
| 1,54-Dibromotetrapentacontane | E | 102 | 545963 | 0 | 0 | 8868529 | 8484580 | 0 | 0 | 0 | 0 | 0 | 0 | 0 | 0 | 0 | 0 | 0 | 0 | 0 | 0 |
| Urea | N | 103 | 1176 | 0 | 0 | 629011 | 789635 | 824426 | 2129818 | 1234312 | 4331378 | 1839928 | 5892993 | 700531 | 608006 | 0 | 5453249 | 4497122 | 19342944 | 3055712 | 4339500 |

**Table S5 |** Weighted average of metabolites’ area under the curve represented as fold change in periapical lesions compared to healthy control.

| Metabolites | Cluster | PubChem ID | Healthy control | Periapical abscess | Radicular cyst | Periapical granuloma |
| --- | --- | --- | --- | --- | --- | --- |
| Palmitic acid | GM | 985 | 39.7 | 9.98 | 36.85 | 8.56 |
| Stearic acid | GM | 5281 | 34.49 | 10 | 29.87 | 13.61 |
| Eicosane | _ | 8222 | 3.32 | 2.55 | 7.05 | 3.64 |
| 2-Bromotetradecane | K | 12798926 | 0.98 | 1.39 | 2.5 | 1.69 |
| Hexadecane | AK | 11006 | 1 | 0.34 | 0.99 | 0.78 |
| Citric acid | AK | 311 | 1.42 | 0.57 | 1.73 | 0.49 |
| Myristic acid | _ | 11005 | 0.78 | 0.41 | 1.46 | 0.58 |
| 2,6,11-Trimethyl dodecane | HN | 35768 | 0.25 | 0.39 | 0.16 | 0.5 |
| Urea | N | 1176 | 0.07 | 0.3 | 0.22 | 0.76 |
| Nonadecane | N | 12401 | 0 | 2.01 | 1.5 | 3.21 |
| 2-Methylhexacosane | N | 150931 | 0 | 1 | 2.01 | 1.64 |
| Disulfide, di-tert-dodecyl | N | 117981 | 0 | 0.38 | 0.62 | 0.9 |
| Oleic acid | F | 445639 | 39.13 | 0 | 0 | 0 |
| 1,54-Dibromo-tetrapentacontane | E | 545963 | 4.78 | 0 | 0 | 0 |
| Pentadecanoic acid | E | 13849 | 3.03 | 0 | 0 | 0 |
| 3-Dodecanol | E | 139108 | 2.39 | 0 | 0 | 0 |
| 2-Hexadecyl-1-eicosanol | D | 86602 | 1.61 | 0 | 0 | 0 |
| 10-Undecynoic acid | D | 31039 | 1 | 0 | 0 | 0 |
| L-Aspartic acid | D | 5960 | 0.71 | 0 | 0 | 0 |
| L-Glutamic acid | D | 33032 | 0.65 | 0 | 0 | 0 |
| 2-Methyl-1,5-heptadiene-3,4-diol | D | 5366259 | 0.41 | 0 | 0 | 0 |
| 2-Butenedioic acid | C | 444972 | 0.24 | 0 | 0 | 0 |
| Itaconic acid | C | 811 | 0.21 | 0 | 0 | 0 |
| Glycolic acid | C | 757 | 0.21 | 0 | 0 | 0 |
| 17-Octadecynoic acid | J | 1449 | 0 | 3.63 | 0 | 0 |
| 4,4-Dimethoxy-2-methyl-2-butanol | I | 4439980 | 0 | 1.85 | 0 | 0 |
| 2,2-Dimethyl-1-octanol | I | 520068 | 0 | 1.62 | 0 | 0 |
| 2-Hydroxy-3-methylbutyric acid | I | 99823 | 0 | 1.43 | 0 | 0 |
| Octacosane | I | 12408 | 0 | 1.32 | 0 | 0 |
| 3-Ethyl-5-(2-ethylbutyl)-octadecane | I | 292285 | 0 | 1 | 0 | 0 |
| Methyl isovalerate | I | 11160 | 0 | 0.99 | 0 | 0 |
| 1-Iodo-tetracosane | _ | 11282694 | 0 | 0.81 | 0 | 0 |
| 10-Methylnonadecane | _ | 530070 | 0 | 0.72 | 0 | 0 |
| 2-Propenoic acid | _ | 6581 | 0 | 0.42 | 0 | 0 |
| 2,2-Dimethyl-1-decanol | _ | 520069 | 0 | 0.38 | 0 | 0 |
| 1-Nonadecene | K | 29075 | 0 | 0 | 5.79 | 0 |
| 5,5-Diethylpentadecane | K | 85977274 | 0 | 0 | 3.92 | 0 |
| Octadecane | K | 11635 | 0 | 0 | 2.84 | 0 |
| Methoxyacetic acid 4-hexadecyl ester | L | 545959 | 0 | 0 | 1.61 | 0 |
| 5-Butyl-nonane | L | 300476 | 0 | 0 | 1.11 | 0 |
| Nonyl tetracosyl ether | L | 87077463 | 0 | 0 | 1.07 | 0 |
| Ethanimidic acid | L | 178 | 0 | 0 | 1 | 0 |
| 8-Methyl-heptadecane | L | 292723 | 0 | 0 | 0.88 | 0 |
| 3-Ethyl-3-methylheptane | L | 140213 | 0 | 0 | 0.85 | 0 |
| Nonadecyl pentafluoropropionate | L | 91693316 | 0 | 0 | 0.84 | 0 |
| 11-Methyldodecanol | L | 33865 | 0 | 0 | 0.64 | 0 |
| Dodecyl nonyl ether | L | 87077689 | 0 | 0 | 0.61 | 0 |
| 1-Piperidinecarboxaldehyde | L | 17429 | 0 | 0 | 0.47 | 0 |
| Beta-Sitosterol | _ | 222284 | 0 | 0 | 0.19 | 0 |
| L-(+)-Lactic acid | P | 107689 | 0 | 0 | 0 | 24.64 |
| Decanoic acid | N | 2969 | 0 | 0 | 0 | 1.69 |
| Petroselinic acid | N | 5281125 | 0 | 0 | 0 | 1.2 |
| Octanoic acid | N | 379 | 0 | 0 | 0 | 1 |
| 6-Ethyl-3-decanol | N | 140584 | 0 | 0 | 0 | 0.63 |
| Sulfurous acid, 2-propyl tridecyl ester | N | 6420355 | 0 | 0 | 0 | 0.39 |
| 2,6,10-Trimethyl-dodecane | _ | 19773 | 0 | 0 | 0 | 0.17 |
| 2,6,10-Trimethyl-tetradecane | _ | 85785 | 0 | 0 | 0 | 0.14 |

**Table S6 |** Pathway and process enrichment analysis in healthy control, periapical abscess, radicular cyst, and periapical granuloma.

| *MCODE | GO | Description | Log10(P) |
| --- | --- | --- | --- |
| **Healthy control** |  |  |  |
| MCODE-1 | R-HSA-438066 | Unblocking of N-methyl-D-aspartate (NMDA) receptors, glutamate binding and activation | -25.5 |
| MCODE-1 | GO:0035235 | ionotropic glutamate receptor signalling pathway | -24.8 |
| MCODE-1 | ko05033 | Nicotine addiction | -23.1 |
| MCODE-2 | R-HSA-416476 | G alpha (q) signalling events | -8.5 |
| MCODE-2 | R-HSA-500792 | G protein coupled receptors ligand binding | -7.1 |
| MCODE-2 | GO:0051480 | regulation of cytosolic calcium ion concentration | -5.1 |
| MCODE-3 | R-HSA-451306 | Ionotropic activity of kainate receptors | -10.2 |
| MCODE-3 | R-HSA-451308 | Activation of Ca-permeable Kainate Receptor | -10.2 |
| MCODE-3 | GO:0035235 | ionotropic glutamate receptor signalling pathway | -9.1 |
| **Periapical abscess** |  |  |  |
| MCODE-1 | R-HSA-2142816 | Synthesis of (16-20)-hydroxyeicosatetraenoic acids (HETE) | -10.6 |
| MCODE-1 | GO:0097267 | Omega-hydroxylase P450 pathway | -10.5 |
| MCODE-1 | GO:0019373 | Epoxygenase P450 pathway | -9.5 |
| **Radicular cyst** |  |  |  |
| MCODE-1 | R-HSA-9619483 | Activation of AMPK downstream of NMDARs | -18.1 |
| MCODE-1 | R-HSA-380972 | Energy dependent regulation of mTOR by LKB1-AMPK | -18.1 |
| MCODE-1 | hsa04710 | Circadian rhythm | -18.0 |
| **Periapical granuloma** |  |  |  |
| MCODE-1 | R-HSA-383280 | Nuclear Receptor transcription pathway | -13.7 |
| MCODE-1 | GO:0006367 | transcription initiation from RNA polymerase II promoter | -10.9 |
| MCODE-1 | GO:0009755 | hormone-mediated signalling pathway | -10.7 |

*The analysis was applied to each MCODE component independently, and the three best-scoring terms by *P*-value were retained as the functional description of the corresponding components.


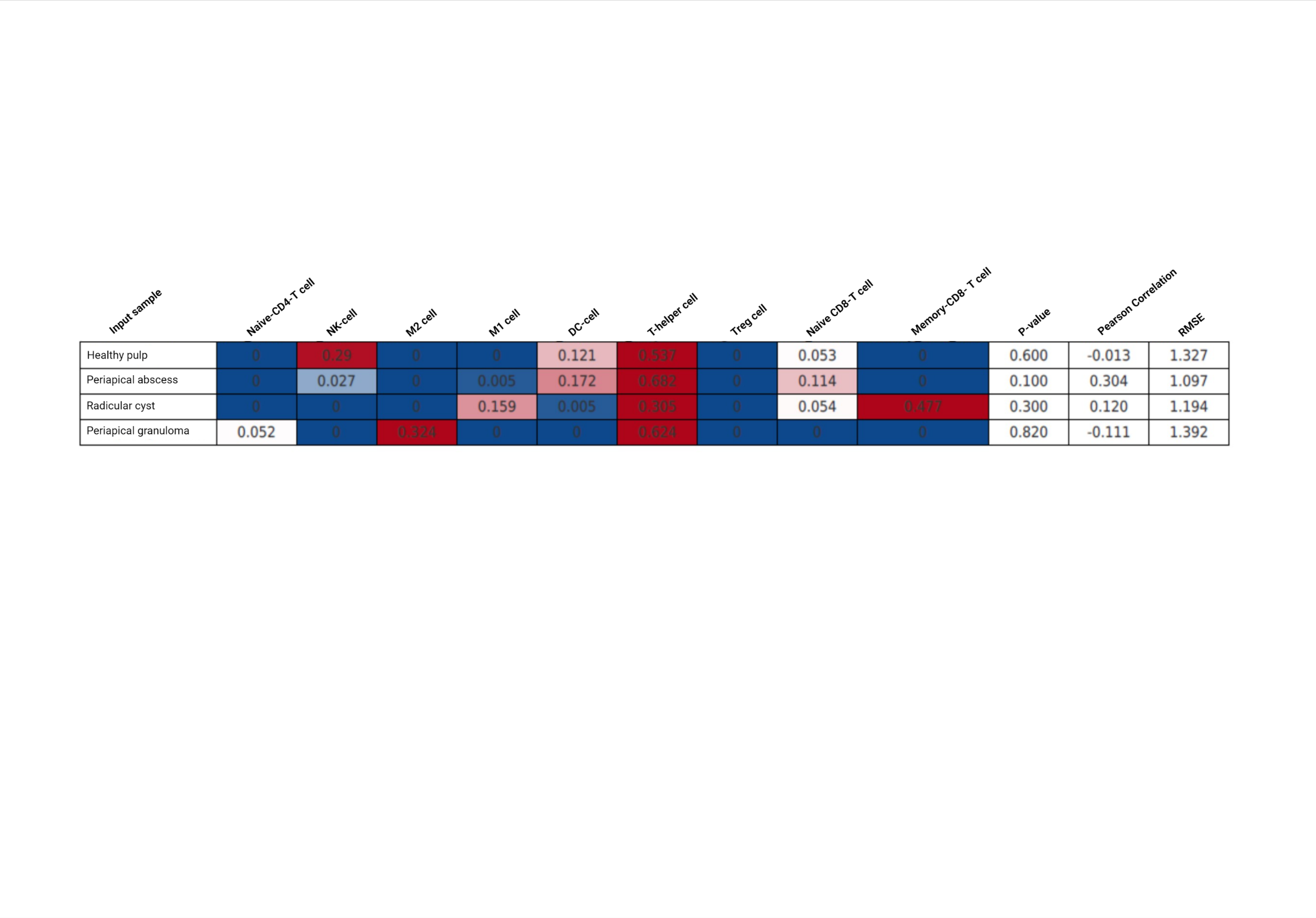
**Table S7 |** *In silico* CIBERSORT values of involved immune cells in healthy pulp tissues and periapical lesions.

**References**

1. Bialesova L, Kulyté A, Petrus P, Sinha I, Laurencikiene J, Zhao C, et al. Epigenetic regulation of PLIN1 in obese women and its relation to lipolysis. *Sci Rep* (2017) 7(1):10152. doi: 10.1038/s41598-017-09232-y.

2. Hanlon MM, Rakovich T, Cunningham CC, Ansboro S, Veale DJ, Fearon U, et al. STAT3 mediates the differential effects of oncostatin M and TNFα on RA synovial fibroblast and endothelial cell function. *Front Immunol* (2019) 10:2056. doi: 10.3389/fimmu.2019.02056.

3. Zhu Y, Schieber EB, McGiff JC, Balazy M. Identification of arachidonate P-450 metabolites in human platelet phospholipids. *Hypertension* (1995) 25(4 Pt 2):854-9. doi: 10.1161/01.hyp.25.4.854.

4. Li X, Wang S, Zhu R, Li H, Han Q, Zhao RC. Lung tumor exosomes induce a pro-inflammatory phenotype in mesenchymal stem cells via NFκB-TLR signaling pathway. *J Hematol Oncol* (2016) 9(1):42. doi: 10.1186/s13045-016-0269-y.

5. Schindler TI, Wagner J-J, Goedicke-Fritz S, Rogosch T, Coccejus V, Laudenbach V, et al. TH17 Cell frequency in peripheral blood is elevated in overweight children without chronic inflammatory diseases. *Front Immunol* (2017) 8:1543. doi: 10.3389/fimmu.2017.01543.

6. Wang S, Li X, Zhao RC. Transcriptome analysis of long noncoding RNAs in toll-like receptor 3-activated mesenchymal stem cells. *Stem Cells Int* (2016) 2016:6205485. doi: 10.1155/2016/6205485.

7. Balint B, Yin H, Nong Z, Arpino JM, O'Neil C, Rogers SR, et al. Seno-destructive smooth muscle cells in the ascending aorta of patients with bicuspid aortic valve disease. *EBioMedicine* (2019) 43:54-66. doi: 10.1016/j.ebiom.2019.04.060.
